# Approximating prediction error variances and reliabilities in a multiple-trait genomic prediction model using Monte Carlo sampling

**DOI:** 10.1007/s00122-026-05336-0

**Published:** 2026-08-28

**Authors:** Antero Heikkilä, Ismo Strandén, Martin H. Lidauer, Klaus Nordhausen, Sara Taskinen

**Affiliations:** 1 Department of Mathematics and Statistics, University of Jyväskylä, 40014 Jyväskylä, Finland; 2 Natural Resources Institute Finland (Luke), 31600 Jokioinen, Finland; 3 Department of Mathematics and Statistics, University of Helsinki, 00014 Helsinki, Finland

**Keywords:** 35A01, 65L10, 65L12, 65L20, 65L70

## Abstract

**Key message:**

We evaluated four existing Monte Carlo-based methods for approximating prediction error variances and reliabilities in a multiple-trait genomic prediction model. All four methods yielded consistent approximations of the exact values.

**Abstract:**

Genomic prediction models, such as genomic best linear unbiased prediction (GBLUP), use genomic data to improve the accuracy of estimated genetic values. As the number of genotypes and traits increases, the exact calculation of prediction error variances (PEVs) and reliabilities becomes computationally infeasible due to the need to invert the coefficient matrix of the mixed model equations, whose dimension increases directly with the number of individuals and traits. The objective of this study was to evaluate the applicability of the Monte Carlo (MC) sampling method to approximate PEVs and reliabilities in a multiple-trait GBLUP framework relevant to hybrid breeding. The MC method avoids direct matrix inversion by repeatedly sampling genetic values from their assumed distributions to approximate PEVs. We applied the MC method using four previously published formulas to approximate PEVs and reliabilities. All formulas produced consistent estimates of PEVs and reliabilities, with convergence rates depending on the formula, the level of reliability, and the MC sample size.

**Supplementary Information:**

The online version contains supplementary material available at https://doi.org/10.1007/s00122-026-05336-0.

## Introduction

The computation of reliabilities for estimated genetic values (EGVs) is often computationally intensive. Reliability, the squared correlation between the true genetic value (*u*) and its estimate ($\hat{u}$) under conceptual repeated sampling, can be expressed in terms of the prediction error variance (PEV) and the genetic variance of *u*. Reliability ranges from 0 to 1 and reflects the amount of information available for predicting genetic value (Mrode and Thompson 2014). Higher reliability indicates greater precision in EGV. PEV is defined as Var(${u}-{\hat{u}}$) (Mrode and Thompson 2014), and its values are obtained from the inverse of the coefficient matrix of the mixed model equations MMEs,, (Henderson, 1975).

Genomic best linear unbiased prediction (GBLUP) is a prediction model that utilizes genomic information from the breeding population. In GBLUP, genetic values are modeled as random effects, with the covariance matrix of genetic values equal to a genomic relationship matrix times genetic variance. The genomic relationship matrix is typically constructed according to the first method by VanRaden (2008). A multiple-trait GBLUP (MT-GBLUP) enables modeling two or more correlated traits.

MT-GBLUP is also appropriate for scenarios involving genotype-by-environment (GxE) interactions. GxE represents the genetic component of phenotypic plasticity, which is the ability of an organism to produce different phenotypes in different environments (de Jong and Bijma 2002). A trait expressed in different environments can be considered a distinct character or character state (Falconer 1952). The MT-GBLUP model incorporates GxE by modeling each character state as a separate trait.

Genomic prediction can be used in hybrid breeding, where additive genetic effects, dominance, and epistasis are incorporated into the genomic prediction model. For example, the model can include general combining ability (GCA) and specific combining ability (SCA), which represents the deviation from parental breeding values (Falconer and Mackay 1996). GCAs model the additive genetic effects that parents pass to progeny. Whereas SCA captures non-additive genetic effects. The genetic value of a cross is the sum of GCAs and SCA (Falconer and Mackay 1996).

Hybrid breeding has been especially popular in maize breeding, where GCAs and SCAs are modeled as separate random effects; see, e.g., Massman et al. (2013) and Technow et al. (2014). When parent lines are fully inbred, the variance of GCAs equals the additive genetic variance in the parent population, while the variance of SCAs captures the non-additive genetic variance (dominance and epistasis) (Falconer and Mackay 1996).

Methods for approximating the accuracy of EGVs, particularly PEVs and reliabilities, have been extensively developed and applied in animal breeding models. Because models such as BLUP, GBLUP, and SNP-BLUP are also applicable to plant breeding (Alemu et al. 2024), these approximation methods are also relevant for plant breeding. However, plant breeding differs from animal breeding in several aspects, including the use of fully inbred lines, hybrid populations, and structured mating designs, all of which influence the data used in these models (Schön and Simianer 2015). Additionally, non-additive genetic effects are of high importance in plant breeding.

García-Cortés et al. (1995) presented a resampling method to approximate PEVs. It is a general method that uses Monte Carlo (MC) sampling of genetic values. It uses the genetic variance and, through repeated sampling from the assumed distribution, compares the simulated values to their predictions. Although this provides consistent approximations of PEVs, it is computationally intensive because the MMEs must be solved multiple times to achieve accurate approximations.

Fouilloux and Laloë (2001) introduced similar MC-based methods to approximate both PEVs and prediction error covariances. Their approach utilizes the empirical covariance structure of the sampled genetic values. Their method is also computationally demanding.

Hickey et al. (2009) developed and evaluated ten MC sampling-based approximation formulas using a single-trait animal model. They observed varying convergence rates across the formulations that are due to differences in asymptotic sampling variances of these methods. According to their results, about 300 MC samples may provide satisfactory convergence, although this requires solving the MMEs the same number of times.

The objective of this study is to demonstrate the application of MC simulation-based methods, introduced by García-Cortés et al. (1995) and Hickey et al. (2009), using an MT-GBLUP model with three random effect groups: heterotic parent pools and hybrids. We emphasize that this study does not introduce new theory, but it evaluates existing methods in a plant breeding framework. The study compares the MC approximated PEVs and reliabilities to exact values, which are obtained by inverting the coefficient matrix of the MMEs. The methods are evaluated using different genetic parameters, including genetic variances, genetic correlations, heritabilities, and varying MC sample sizes. We define and introduce the MT-GBLUP model in the Materials and methods section. Then, we introduce the MC-based method for approximating PEVs and reliabilities. The Results section presents the performance of the approximation method. Finally, we discuss and conclude our findings.

## Materials and methods

### Genomic prediction models

We consider a multivariate linear mixed model with *H* random effect groups, where the response variables correspond to different traits or character states. The model is presented in vectorized form as follows:

1 $$\begin{aligned} \textbf{y} = \textbf{Xb} + \sum _{h=1}^{H} \textbf{W}_h \textbf{u}_h + \textbf{e}, \end{aligned}$$

where $$\textbf{y} = \begin{bmatrix} \textbf{y}_1^T ,\dots , \textbf{y}_k^T \end{bmatrix}^T$$ is an N×1 vector of phenotypes across *k* traits, with $N= \sum _{t=1}^{k}N_t$, and $N_t$ denoting the number of observations for trait *t*, with t=1,⋯,k.

Let

$$\begin{aligned} \textbf{X} = \begin{bmatrix} \textbf{X}_1 & \dots & \boldsymbol{0} \\ \vdots & \ddots & \vdots \\ \boldsymbol{0}& \dots & \textbf{X}_k \end{bmatrix}\end{aligned}$$

denote the N×f block-diagonal design matrix for fixed effects, where each block $\textbf{X}_t$ is the design matrix for trait *t*, and $f=\sum _{t=1}^{k} f_t$ is the total number of fixed effects across all traits. The corresponding fixed effects coefficients are denoted as $$\textbf{b} = \begin{bmatrix} \textbf{b}_1^T, \dots , \textbf{b}_k^T \end{bmatrix}^T$$, which is an f×1 vector, where $\textbf{b}_t$ is the vector of fixed effects for trait *t*.

Let

$$\begin{aligned} \textbf{W}_h= \begin{bmatrix} \textbf{W}_{h,1} & \dots & \boldsymbol{0} \\ \vdots & \ddots & \vdots \\ \boldsymbol{0}& \dots & \textbf{W}_{h,k} \end{bmatrix} \end{aligned}$$

denote the $N \times (kn_h)$ block-diagonal incidence matrix for random effect group *h*, where each block $\textbf{W}_{h,t}$ is the incidence matrix for trait *t*, and $n_h$ is the number of levels in the random effect group *h*. The corresponding random effects are denoted by $$\textbf{u}_h = \begin{bmatrix} \textbf{u}_{h,1}^T, \dots , \textbf{u}_{h,k}^T \end{bmatrix}^T$$, a $(kn_h) \times 1$ vector, where each $\textbf{u}_{h,t}$ is the vector of random effects for trait *t*. Here, $n_h$ can be regarded as the number of individuals in the random effect group *h*. We assume that $n_h$ is the same for all traits.

The random effects are assumed to follow a multivariate normal (MVN) distribution: $\textbf{u}_h \sim \text {MVN}(\boldsymbol{0}, \textbf{G}_{\textbf{0},h} \otimes \textbf{G}_h)$, where $\textbf{G}_h$ is an $n_h \times n_h$ genomic relationship matrix for random effect group *h*. The matrix $\textbf{G}_{\textbf{0},h}$ is a k×k genetic covariance matrix across traits for group *h*, with trait-specific genetic variances on the diagonal and covariances on the off-diagonal.

The residual vector $$\textbf{e} = \begin{bmatrix} \textbf{e}_1^T, \dots , \textbf{e}_k^T \end{bmatrix}^T$$ is assumed to follow $\textbf{e} \sim \text {MVN}(\boldsymbol{0}, \textbf{R})$, where $\textbf{R}$ is an N×N covariance matrix. For simplicity of presentation, we assume that $\textbf{R} = \mathbf {R_0} \otimes \textbf{I}$, i.e., all traits are observed or not in a residually correlated block. The matrix $\mathbf {R_0}$ is a k×k residual covariance matrix across traits, with residual variances on the diagonal and covariances on the off-diagonal. The identity matrix is denoted as $\textbf{I}$. Random effects and residuals are assumed to be uncorrelated.

We assume that the variance components (VCs), including genetic and residual (co)variances, are known. Therefore, the matrices $\textbf{G}_{\textbf{0},h}$ and $\textbf{R}$ are known.

For example, an MT-GBLUP model with H=3 random effect groups is expressed as follows:

2 $$\begin{aligned} \textbf{y} = \textbf{Xb} + \textbf{W}_1 \textbf{u}_1 +\textbf{W}_2 \textbf{u}_2 + \textbf{W}_3 \textbf{u}_3 + \textbf{e}. \end{aligned}$$

The MMEs to obtain the estimated fixed effects and the predicted random effects of Eq. (2) are given in Eq. (3).

3 $$\begin{aligned}&\begin{bmatrix} \textbf{X}^T\textbf{R}^{-1}\textbf{X} & \textbf{X}^T\textbf{R}^{-1}\textbf{W}_1 & \textbf{X}^T\textbf{R}^{-1}\textbf{W}_2 & \textbf{X}^T\textbf{R}^{-1}\textbf{W}_3 \\ \textbf{W}_1^T\textbf{R}^{-1}\textbf{X} & \textbf{W}_1^T\textbf{R}^{-1}\textbf{W}_1 + \textbf{G}_{\textbf{0},1}^{-1} \otimes \textbf{G}_{1}^{-1} & \textbf{W}_1^T\textbf{R}^{-1}\textbf{W}_2 & \textbf{W}_1^T\textbf{R}^{-1}\textbf{W}_3 \\ \textbf{W}_2^T\textbf{R}^{-1}\textbf{X} & \textbf{W}_2^T\textbf{R}^{-1}\textbf{W}_1 & \textbf{W}_2^T\textbf{R}^{-1}\textbf{W}_2 +\textbf{G}_{\textbf{0},2}^{-1} \otimes \textbf{G}_{2}^{-1} & \textbf{W}_2^T\textbf{R}^{-1}\textbf{W}_3 \\ \textbf{W}_3^T\textbf{R}^{-1}\textbf{X} & \textbf{W}_3^T\textbf{R}^{-1}\textbf{W}_1 & \textbf{W}_3^T\textbf{R}^{-1}\textbf{W}_2 & \textbf{W}_3^T\textbf{R}^{-1}\textbf{W}_3 + \textbf{G}_{\textbf{0},3}^{-1} \otimes \textbf{G}_{3}^{-1} \end{bmatrix} \begin{bmatrix} \hat{\textbf{b}} \\ \hat{\textbf{u}}_\text {1} \\ \hat{\textbf{u}}_\text {2} \\ \hat{\textbf{u}}_\text {3} \end{bmatrix} = \begin{bmatrix} \textbf{X}^T\textbf{R}^{-1}\textbf{y} \\ \textbf{W}_1^T\textbf{R}^{-1}\textbf{y} \\ \textbf{W}_2^T\textbf{R}^{-1}\textbf{y} \\ \textbf{W}_3^T\textbf{R}^{-1}\textbf{y} \end{bmatrix}. \end{aligned}$$

### Prediction error variance and reliability

In the context of MMEs, PEV can be derived from the inverse of the coefficient matrix $\textbf{C}$ (Henderson 1975). For a random effect group *h*, PEV is obtained as:

4 $$\begin{aligned} \textbf{PEV}_h = \text {diag}(\textbf{C}^{hh}), \end{aligned}$$

with dimension $(kn_h) \times 1$. $\textbf{C}^{hh}$ denotes the block of $\textbf{C}^{-1}$ corresponding to random effect group *h*.

The reliability for individual *i* in group *h* and trait *t* can be calculated as (Van Vleck 1993):

5 $$\begin{aligned} r_{i,h,t}^2 = 1 - \frac{\text {PEV}_{h,t,i}}{ \text {[} \textbf{G}_h\text {]}_{ii} \sigma ^2_{t,h}}, \end{aligned}$$

where $\text {PEV}_{h,t,i}$ is the PEV for individual *i* in group *h* and trait *t*, $[ \textbf{G}_{h} ]_{ii}$ is the *i*th diagonal element of the genomic relationship matrix for group *h*, and $\sigma ^2_{t,h}$ is the genetic variance for trait *t* in group *h*.

### Multiple-trait model for hybrids

We apply a multiple-trait genomic model in plant breeding, including fixed effects and three random effect groups: the GCAs of both parents, and the SCA of their cross.

We consider a multivariate setting with two traits evaluated in two environments, resulting in four character states treated as distinct response variables. Phenotypic observations are made on hybrids, which are crosses between inbred lines from two heterotic parent pools, referred to as parent pool 1 and parent pool 2. Here, $\textbf{u}_1$ and $\textbf{u}_2$ denote the GCA effects of parents from pools 1 and 2, respectively, while $\textbf{u}_3$ denotes the SCA effects of their crosses. This setting corresponds directly to Eq. (2).

The genomic relationship matrix $\textbf{G}$, describing the genetic relatedness among genotyped individuals, can be constructed from SNP data following method 1 of VanRaden (2008):

6 $$\begin{aligned} \textbf{G}= \frac{\textbf{ZZ}^T}{2\sum _{j=1}^m p_j(1-p_j)}, \end{aligned}$$

where $\textbf{Z}$ is an $n_{G} \times m$ matrix of centered SNP marker genotypes, *m* is the number of SNP markers, $n_G$ is the number of genotyped individuals, and $p_j$ is the frequency of the second allele at locus *j*. A normalized genomic relationship matrix $(\mathbf {G^*})$ can be obtained by rescaling $\textbf{G}$ to have average diagonal coefficients equal to 1 (Forni et al. 2011):

7 $$\begin{aligned} \mathbf {G^*} = d \ \textbf{G}, \ \text {where } d=\frac{n_G}{\text {tr}(\textbf{G})}. \end{aligned}$$

As the genomic relationship matrix is often singular, it is common to blend it by adding a small value to the diagonal, resulting in $\mathbf {G_b} = (1-b) \textbf{G} + b \textbf{I}$, where *b* is a chosen, typically small value (e.g., 0.01).

We constructed the normalized genomic relationship matrices $\textbf{G}_1^*$ and $\textbf{G}_2^*$ for the parent groups using Eqs. (6) and (7). The genomic relationship matrix of all possible crosses is defined as $\textbf{G}_{\text {cross}} = \textbf{G}_1 \otimes \textbf{G}_2$. The submatrix of $\textbf{G}_{\text {cross}}$ corresponding to the crosses that were actually realized is denoted as $\textbf{G}_3$ and its normalized version is denoted as $\textbf{G}_3^*$. To ensure invertibility, we blended each matrix using b=0.01 times an identity matrix.

We assumed the following distributions for the genetic values: $\textbf{u}_1 \sim \text {MVN}(\boldsymbol{0}, \textbf{G}_{\textbf{0},1} \otimes \textbf{G}_1^*)$, $\textbf{u}_2 \sim \text {MVN}(\boldsymbol{0}, \textbf{G}_{\textbf{0},2} \otimes \textbf{G}_2^*)$, and $\textbf{u}_3 \sim \text {MVN}(\boldsymbol{0}, \textbf{G}_{\textbf{0},3} \otimes \textbf{G}_3^*)$. By setting up the MMEs as in Eq. (3), where the genomic relationship matrices $\textbf{G}_h$ are replaced with normalized and blended versions $\textbf{G}_h^*$, we obtain the exact PEVs and reliabilities using Eqs. (4) and (5).

### MC sampling for approximating PEVs

MC-based simulation methods approximate PEVs by simulating multiple samples from assumed distributions of genetic values. We used a method based on García-Cortés et al. (1995) and Hickey et al. (2009). It consists of three steps: i) simulating genetic values, residuals, and phenotypes, ii) solving the MMEs emerging from the simulated data, and iii) using formulas involving simulated true and estimated genetic values in approximating PEVs.

In order for the MC-based method to be faster than the exact calculation of PEVs, solving of MMEs should be done with a fast iterative solver, such as the preconditioned conjugate gradient (PCG) algorithm. The principle of the PCG algorithm is presented, e.g., in Tsuruta et al. (2001).

We assume that the VCs, including genetic and residual (co)variances, are known. Here, we describe the PEV approximation process in the MT-GBLUP context, following García-Cortés et al. (1995) and Hickey et al. (2009).

### PEV approximation algorithm

Algorithm 1 presents the MC method used to approximate PEVs. The VCs, marker matrices, and the model are assumed to be known. VCs of genomic models can be efficiently estimated, e.g., using the augmented average information restricted maximum likelihood (AI-REML) algorithm presented in Strandén et al. (2024).

The simulated genetic values ${\tilde{\textbf{u}}}_{h}$ can be generated in two ways: either by using the marker matrices or by using Cholesky decompositions of genomic relationship matrices. Algorithm 1 presents the simulation via centered marker matrices $\textbf{Z}_h$.

The centered marker matrices can be obtained directly from the original marker matrices. In the MT-GBLUP model for hybrids, the marker matrices of the parents are known, and the construction of $\textbf{Z}_1$ and $\textbf{Z}_2$ is straightforward. The centered SCA interaction marker matrix $\textbf{Z}_3$ can be obtained from the centered marker matrices of the parents.

As $\textbf{G}_{\text {cross}} = \textbf{G}_1 \otimes \textbf{G}_2 = (\textbf{Z}_1 \otimes \textbf{Z}_2)(\textbf{Z}_1 \otimes \textbf{Z}_2)^T$ (up to scaling), the exact full centered SCA interaction marker matrix for all possible hybrids would be $\textbf{Z}_1 \otimes \textbf{Z}_2$ with dimensions $n_1n_2 \times m^2$. To avoid forming this large matrix, we instead used a computationally lighter construction $\textbf{Z}_{\text {cross}} = (\textbf{Z}_1 \otimes \textbf{1}_{n_2}) \circ (\textbf{1}_{n_1} \otimes \textbf{Z}_2),$ with dimensions $n_1n_2 \times m$, where $\textbf{1}_{n_1}$ and $\textbf{1}_{n_2}$ denote vectors of ones of lengths $n_1$ and $n_2$, respectively, and ∘ denotes the Hadamard product. Using this construction $\textbf{Z}_{\text {cross}} \textbf{Z}_{\text {cross}} ^T \approx \textbf{G}_1 \otimes \textbf{G}_2$. We verified empirically that this approximation does not affect the validity of the method (see Section Results for more details).

Often, only a subset of all possible crosses is relevant. The corresponding submatrix of $\textbf{Z}_{\text {cross}}$ can be constructed by selecting the appropriate rows from the centered marker matrices $\textbf{Z}_1$ and $\textbf{Z}_2$. Specifically, for hybrid *i*, $[\textbf{Z}_3]_{i\cdot } = [\textbf{Z}_1]_{p_1\cdot } \circ [\textbf{Z}_2]_{p_2\cdot },$ where $[\textbf{Z}_3]_{i\cdot }$ denotes the *i*th row of $\textbf{Z}_3$, and $p_1$ and $p_2$ index the two parents of hybrid *i*.

An alternative strategy for simulating genetic values is to use Cholesky decompositions of the genomic relationship matrices: ${\tilde{\textbf{u}}}_{h} = \text {vec}( \textbf{L}_{h}\textbf{S}_h {\textbf{L}^T_{{{\textbf{0},h}}}})$, where $\textbf{L}_h$ and $\textbf{L}_{\textbf{0},h}$ are the Cholesky factors of $\textbf{G}_h^*$ and $\textbf{G}_{\textbf{0},h}$, respectively, and the elements of $\textbf{S}_h$ are simulated from N(0,1).

The simulation strategy chosen for genetic values affects steps 1–6 in Algorithm 1. Henceforth, we refer to simulations via centered marker matrices as Marker-based MC, and simulations via Cholesky decompositions as Cholesky MC.

The number of multiplications needed to simulate the genetic values $\tilde{\textbf{u}}_{h}$ depends on the strategy. Assuming $k \ll \text {min}(m,n_G)$, where *k* denotes the number of traits, the dominant computations require $n_Gmk$ and $\frac{1}{2}n_G(n_G+1)k$ multiplications for Marker-based MC ($\textbf{Z}_h\textbf{V}_h$) and Cholesky MC ($\textbf{L}_{h}\textbf{S}_h {\textbf{L}^T_{{{\textbf{0},h}}}}$), respectively. Thus, the number of computations is greater in Cholesky MC when $n_G \ge 2m$. Additionally, Cholesky MC requires $\mathcal {O}(n_G^3)$ computations to compute $\textbf{L}_h$, while Marker-based MC requires $\mathcal {O}(n_Gm)$ computations for $\text {tr}(\textbf{Z}_h\textbf{Z}_h^T)$. The overall computational complexities are thus $\mathcal {O}(n_Gm) +\mathcal {O}( n_{\text {mc}} n_Gmk)$ and $\mathcal {O}(n_G^3) +\mathcal {O}( n_{\text {mc}} n_G^2k)$ for Marker-based MC and Cholesky MC, respectively.

Because the expected value of the fixed effects does not affect the distribution of random effects (García-Cortés et al. 1995), fixed effects are not simulated in Algorithm 1.

Assuming known VCs, Algorithm 1 is applicable to an MT-GBLUP model with *H* random effect groups, as presented in Eq. (1).

**Figure:**
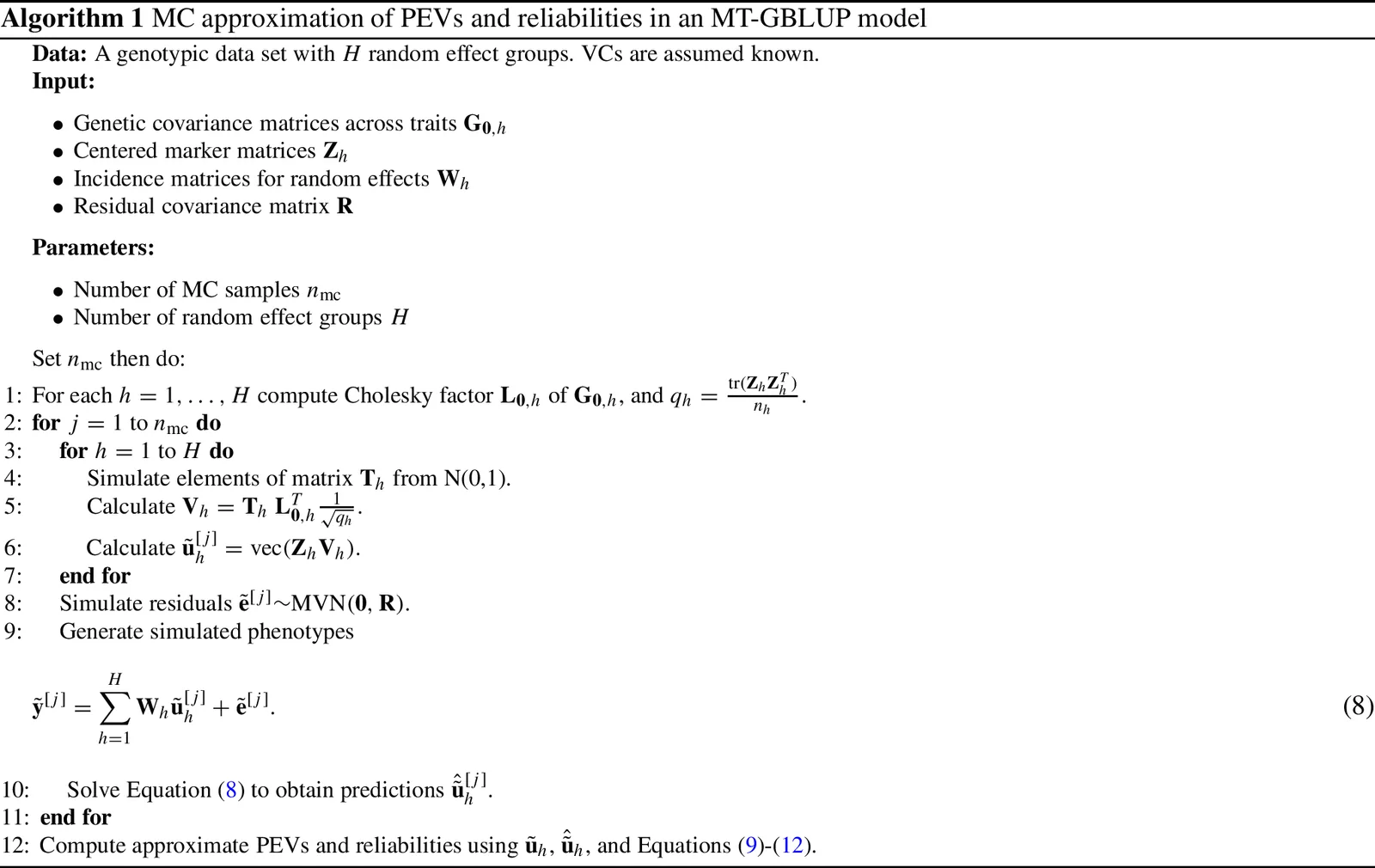

#### PEV approximation formulas

We evaluated four previously published PEV approximation formulas. The first three, called PEV1, PEV2, and PEV3, were introduced by García-Cortés et al. (1995), and the fourth (NF2) was proposed by Hickey et al. (2009).

These formulas compute individual-level and trait-specific PEVs for predicted genetic values $\hat{u}_{i, h, t}$. In the formulas, $[ \textbf{G}_{h}^* ]_{ii}$ is the *i*th diagonal element of the corresponding normalized genomic relationship matrix, and $\sigma _{t,h}^2$ is the genetic variance for trait *t* in group *h*. The empirical variances of PEV1 and PEV2 approximations across multiple MC iterations are denoted as $\text{ Var }(\text{ PEV1 }({{\hat{ u}}}_{i,h,t}))$ and $\text{ Var }(\text{ PEV2 }({{\hat{ u}}}_{i,h,t}))$. Reliability is denoted by $r^2$.

The PEV1 and PEV2 methods are defined as follows:

9 $$\begin{aligned} \text {PEV1}(\hat{ u}_{i,h,t}) = [\textbf{G}_h^*]_{ii} \sigma _{t,h}^2 - \dfrac{\hat{\tilde{\textbf{u}}}_{i,h,t}^T {\hat{\tilde{\textbf{u}}}}_{i,h,t}}{n_{\text {mc}}} \end{aligned}$$

and

10 $$\begin{aligned} \text {PEV2}(\hat{ u}_{i,h,t}) = \dfrac{(\tilde{{\textbf{u}}}_{i,h,t} - {\hat{\tilde{\textbf{u}}}}_{i,h,t})^T(\tilde{{\textbf{u}}}_{i,h,t} - {\hat{\tilde{\textbf{u}}}}_{i,h,t})}{n_{\text {mc}}}. \end{aligned}$$

The PEV3 method is a weighted sum of PEV1 and PEV2 based on their empirical variances:

11 $$\begin{aligned} \text {PEV3}(\hat{ u}_{i,h,t}) = \dfrac{w_1 \text {PEV1}(\hat{ u}_{i,h,t}) + w_2 \text {PEV2}(\hat{ u}_{i,h,t})}{w_1 + w_2}, \end{aligned}$$

where $$w_1 = ({\text {Var}(\text {PEV1}({{\hat{ u}}}_{i,h,t}))})^{-1}$$, and $$w_2 = (\text {Var}(\text {PEV2}({{\hat{ u}}}_{i,h,t})))^{-1}$$.

The NF2 method is defined as:

12 $$\begin{aligned} \text {NF2}(\hat{ u}_{i,h,t}) = \dfrac{\text {PEV2}({ {\hat{ u}}}_{i,h,t})[\textbf{G}_h^*]_{ii} \sigma _{t,h}^2}{\text {PEV2}({{\hat{ u}}}_{i,h,t}) + \text {Var}({\hat{\tilde{\textbf{u}}}}_{i,h,t})}. \end{aligned}$$

The asymptotic sampling variances of all four methods were introduced in Hickey et al. (2009), and are presented in Table 1.

**Table 1:** Asymptotic sampling variances of four methods approximating PEVs as presented in Hickey et al. (2009)

Method	Asymptotic sampling variance
PEV1	$\dfrac{2r^4\sigma _{t,h}^4}{n_\text {mc}}$
PEV2	$\dfrac{2(1-r^2)^2\sigma _{t,h}^4}{n_\text {mc}}$
PEV3	$\dfrac{2r^4(1-r^2)^2}{(1-r^2)^2+r^4} \dfrac{\sigma _{t,h}^4}{n_\text {mc}}$
NF2	$\dfrac{4r^4(1-r^2)^2 \sigma _{t,h}^4}{n_\text {mc}}$

### Study design

We evaluated the behavior of four PEV approximation formulas by comparing the exact PEVs and reliabilities to their approximations. We used a four-trait GBLUP model.

### Data

We used simulated data sets in order to control and define parameters such as genetic variances. Even though the data are simulated, we refer to it as our real data to distinguish it from the data generated during the MC simulation. To mimic hybrid crop breeding, we first created two heterotic parent groups, called parent groups 1 and 2.

We simulated the genotypes of the parent groups using AlphaSimR (Gaynor et al. 2021), and selected maize as the species. The genome consisted of ten chromosomes, and we set the number of segregating sites per chromosome to be 10,000. The number of SNP markers per chromosome was 5000, resulting in a total of 50,000 SNP markers. After that, two founder groups of size 100 were generated, and to mimic the genetic separation of heterotic pools, a population split was introduced 100 generations ago.

Within these two founder populations, parental populations were created by random mating followed by double haploid (DH) production. This yielded two parental populations, each having 1000 individuals. These DH parent populations were then used to generate 10, 000 F1 hybrids by randomly crossing individuals from the two parent groups. The mating design was a balanced and random set of crosses between the two parental populations.

As a result, the data had two parent groups: parent group 1 and 2, with sizes of 1000, and their crosses. The hybrid group consisted of 10,000 individuals. Phenotypic records were made only for the crosses. The data set consisted of four character states (also referred to as traits or phenotypes): two traits measured in two different locations. The traits and locations were assumed to be correlated. Each cross had ten observations per trait, and 10% of the observations were randomly set as missing. Although missing data affects the magnitude of PEV and reliability values, it does not affect the validity of the PEV approximation methods. We assumed a simple fixed effects structure, where each trait had one discrete and one continuous fixed effect, leading to a total of eight fixed effects.

In total, we predicted 48,000 genetic values and computed their exact and approximate PEVs and reliabilities.

### Simulation parameters

The simulation parameters were chosen to mimic a case of two correlated traits measured at two locations. We can imagine that these traits mimic grain yield and disease resistance traits. For trait A, the narrow-sense heritability was set to $\frac{1+1}{2+0.10+5} \approx 0.28$ and the correlation between the two locations was set to 0.80. For trait B, the narrow-sense heritability was $\frac{1+1}{2+0.10+10} \approx 0.17$ and the correlation was 0.70. The traits were assumed to be slightly negatively correlated. The SCA variance was set to 0.10, meaning that 5% of the genetic effects are non-additive. The genetic covariance structure was assumed to be the same for both parental GCA effects.

Trait A mimics the grain yield of maize, whose narrow-sense heritability, according to Hallauer et al. (2010, p. 171), is around 20%. Trait B mimics a disease resistance trait, which is usually negatively correlated with grain yield (Derbyshire et al. 2024). The two environments are assumed to have a strong positive correlation in both traits.

These assumptions lead to the following genetic covariance matrices:

$$\begin{aligned} \textbf{G}_{\textbf{0},1}&=\textbf{G}_{\textbf{0},2} =\begin{bmatrix} 1 & 0.80 & -0.30 & -0.20 \\ 0.80 & 1 & -0.20 & -0.30 \\ -0.30 & -0.20 & 1 & 0.70 \\ -0.20 & -0.30 & 0.70 & 1 \end{bmatrix} \text { and } \\ \textbf{G}_{\textbf{0},3}&=\begin{bmatrix} 0.10 & 0.08 & -0.03 & -0.02 \\ 0.08 & 0.10 & -0.02 & -0.03 \\ -0.03 & -0.02 & 0.10 & 0.07 \\ -0.02 & -0.03 & 0.07 & 0.10 \end{bmatrix}. \end{aligned}$$

Residuals were assumed uncorrelated across character states, yielding the residual covariance matrix:

$$\begin{aligned} \mathbf {R_0}=\begin{bmatrix} 5 & 0 & 0 & 0 \\ 0 & 5 & 0 & 0 \\ 0 & 0 & 10 & 0 \\ 0 & 0 & 0 & 10 \end{bmatrix}. \end{aligned}$$

In both the genetic and residual covariance matrices, the first two rows and columns correspond to trait A in the two locations, and the last two to trait B in the two locations. Off-diagonal elements denote covariances.

### Exact PEVs and reliabilities

We calculated the exact PEVs and reliabilities of the genetic values of Eq. (2) using Eqs. (4) and (5).

### Approximate PEVs and reliabilities

We approximated PEVs using the MC method and the approximation formulas presented in Eqs. (9)–(12). We then estimated reliabilities using Eq. (5), where the exact PEVs were substituted with their approximated ones.

### Comparison of the MC methods

We evaluated the performance of the MC methods using the following statistics: Pearson’s correlation coefficient between the exact and their approximated values, root mean squared error (RMSE), maximum absolute difference between the exact and its approximated values (MAX), and linear model coefficients from the regression, where the dependent variable is the exact value and the independent variable is its approximated value.

We present the results in a reliability scale rather than in the PEV scale, because the scale of PEV depends on the value of the genetic variance and the diagonal elements of the genomic relationship matrix. Using reliability ensures that the results are comparable between groups with different genetic variances. However, results in PEV scale are provided in Supplementary materials 1 and 2.

As the chosen simulation parameters yield only reliabilities in narrow intervals, we ran the simulation with a different set of simulation parameters. Supplementary material 2 presents the results for that setup.

### Software, computational resources and benchmark tests

We evaluated the computational demand of the approximation procedure by comparing the exact method with the MC-based approximation method. In the exact approach, the most time-consuming part is inverting the coefficient matrix. In the MC approximation method, the repeated solving of the MMEs across MC replicates is the main bottleneck, even when using an efficient iterative solver such as PCG. In both the exact MT-GBLUP and MC approaches, one must construct the genomic relationship matrices and their inverses, as well as all other matrices involved in the MMEs.

For the exact method, we present the time needed to invert the coefficient matrix of Eq. (3) and the matrix multiplication with the inverse and the right-hand side of the equation. As the coefficient matrix is symmetric and positive-definite, we used Cholesky inversion in R (R Core Team 2024). For the approximation method, we report the time required to generate $n_{\text {mc}}$ samples for each random effect (steps 1–9 in Algorithm 1) and the time needed to predict random effects once using the PCG algorithm (step 10). Because genetic values can be simulated either by Marker-based MC or Cholesky MC, we report timings for both strategies.

Depending on the number of MC samples, the PCG algorithm has to be applied as many times. However, the computational burden can be mitigated by using parallel computing, where each processor simulates and solves a specified number of MC iterations independently, and finally, the results are combined.

For the PCG step, we used Python with SciPy *scipy.sparse.linalg.cg*,, (Virtanen et al., 2020). We set the relative and absolute tolerances of the PCG to 0.00001. We performed the computation using the CSC Puhti supercomputer using one node with 10 cores and a maximum RAM memory of 1400 GB (CSC - IT Center for Science, Finland 2026). Each node has two Intel Xeon Gold 6230 processors (Cascade Lake, 20 cores, 2.1 GHz).

## Results

The functionality of the MC method for approximating PEVs and reliabilities was demonstrated in an MT-GBLUP framework. All four methods produced consistent approximations of the exact PEVs and reliabilities. However, there were differences in convergence rates. Most of the differences can be explained by different asymptotic sampling variances. The size of the sampling variance is determined by the reliability of the EGV. Low and high reliabilities were approximated well with different formulas. The results were in agreement with the results presented by Hickey et al. (2009).

**Fig. 1:**
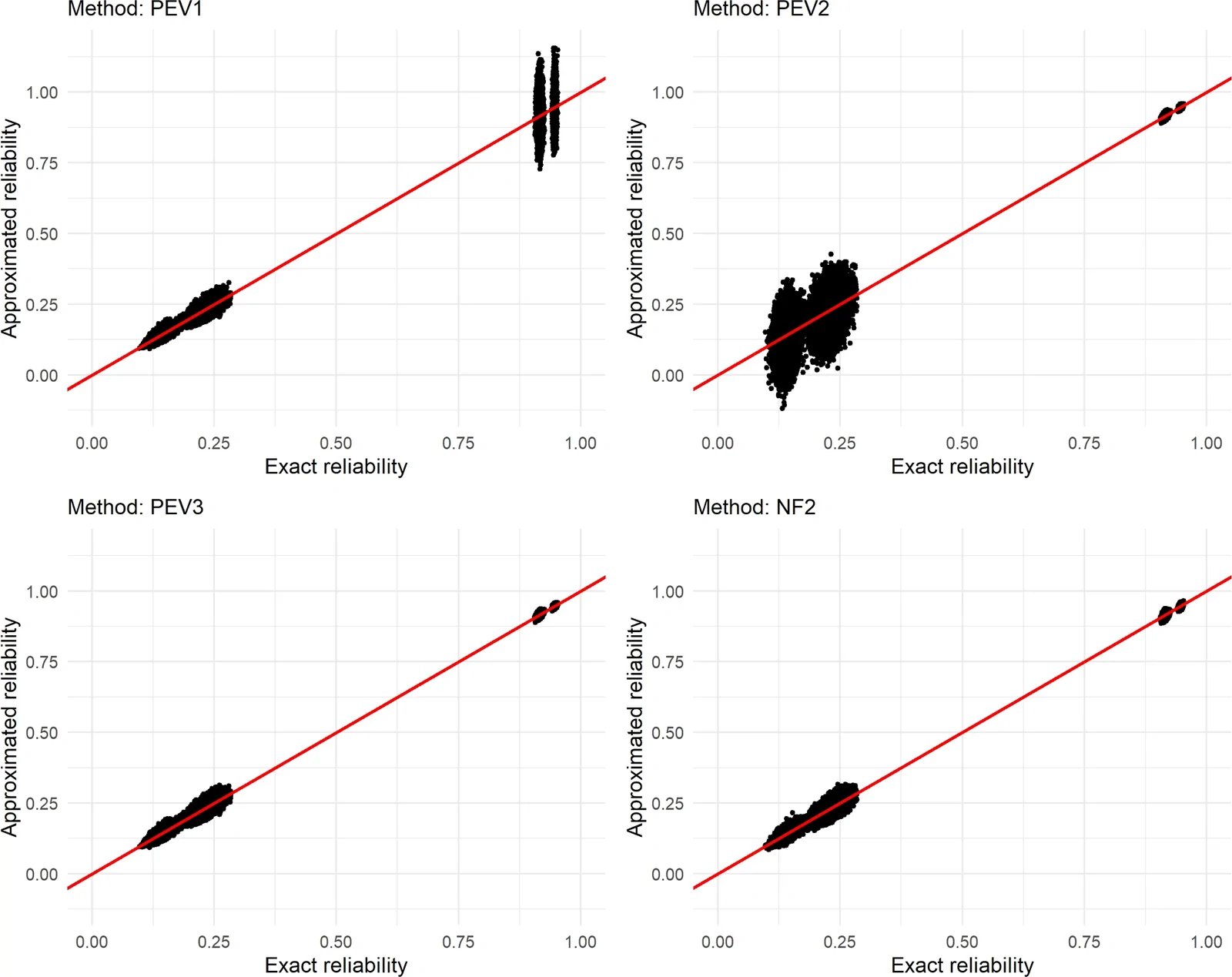
Scatterplots with exact reliability on the x-axis and approximated reliability on the y-axis. Number of MC samples: 500. The red line represents y=x

Table 2 presents statistics for the approximate reliabilities when using 500 MC samples. The correlations between the exact and their approximated reliabilities were over 0.99, except with PEV2. RMSEs were at a maximum of 0.047, MAXs varied between 0.055 and 0.251, and intercepts were close to 0 and slopes close to 1. Figure 1 presents scatter plots for the exact and the approximate reliabilities. Visually, it appears that PEV1 approximates better lower reliabilities and PEV2 higher reliabilities. PEV3 and NF2 seem to provide more stable approximations.

**Table 2:** Comparison of different methods for approximating exact reliability and their estimates with different statistics using 500 MC samples

Statistic	PEV1	PEV2	PEV3	NF2
Correlation	0.996	0.986	0.999	0.999
RMSE	0.026	0.047	0.011	0.013
MAX	0.223	0.251	0.055	0.070
Intercept	0.002	0.008	0.000	0.000
Slope	0.993	0.973	0.999	0.999

Table 3 presents statistics for the approximate reliabilities, but here the statistics are applied to each group and trait combination separately. As the range of reliability in each group-trait combination is narrower than in full-scale reliability, the correlations were lower, and the intercepts and slopes were further apart from 0 and 1, respectively. The narrow range of reliability affected these statistics; thus, RMSE and MAX were more appropriate measures for comparisons. As shown in Supplementary material 1, these statistics still converged to their target values as the number of MC samples increased.

**Fig. 2:**
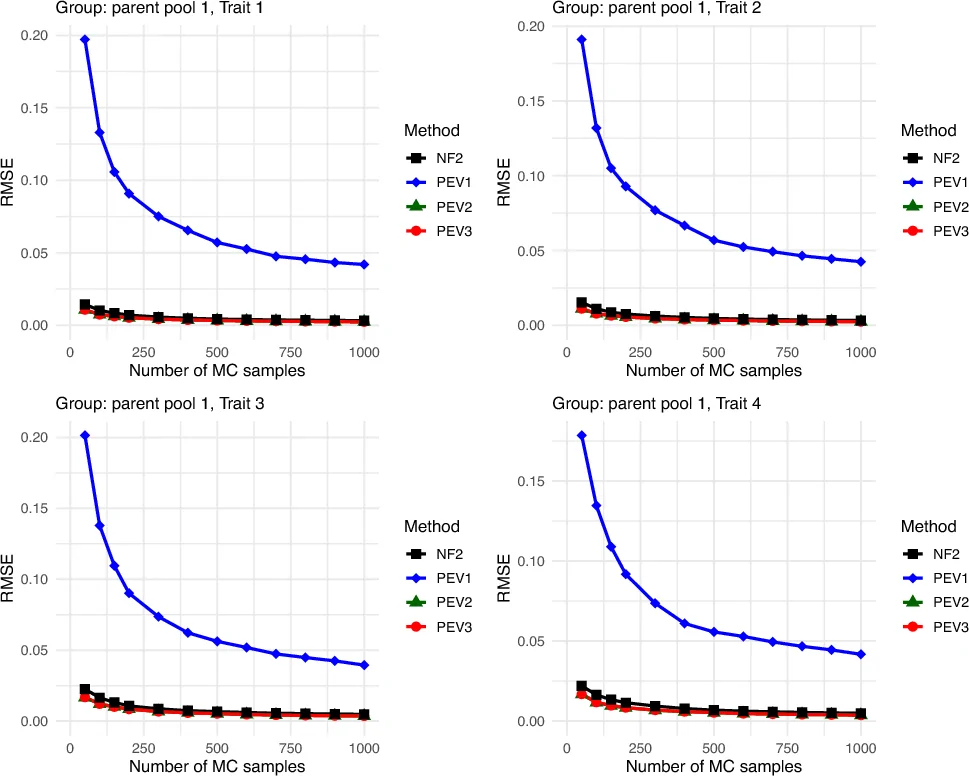
RMSEs of four methods approximating exact reliability using different MC sample sizes (Parent pool 1). PEV2, PEV3, and NF2 show similar trends

**Fig. 3:**
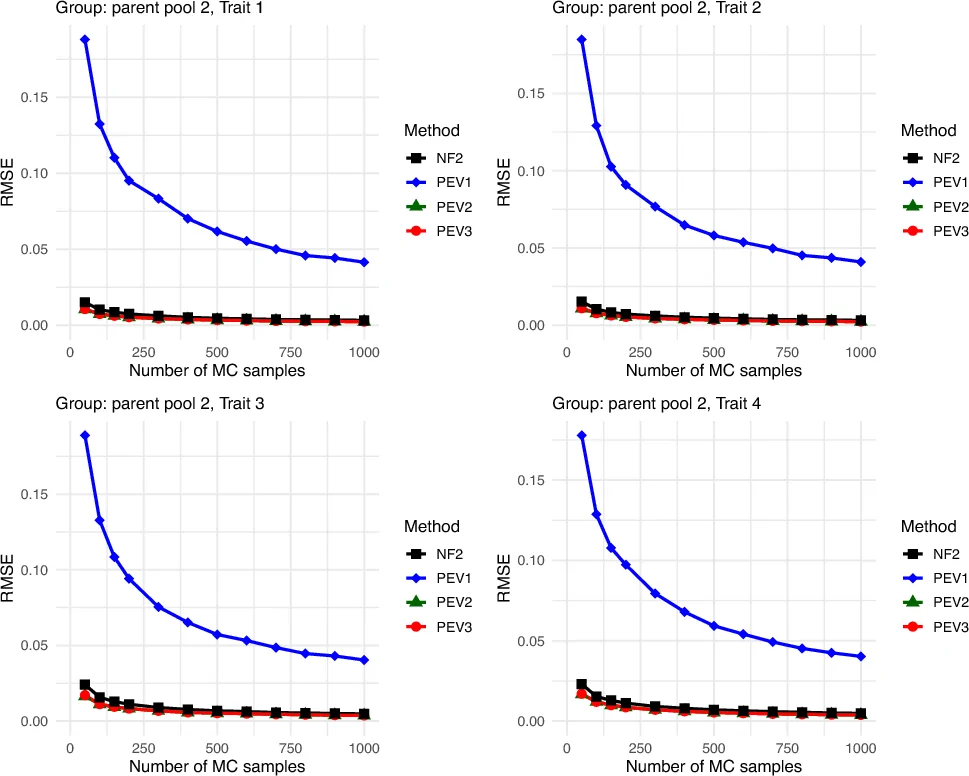
RMSEs of four methods approximating exact reliability using different MC sample sizes (Parent pool 2). PEV2, PEV3, and NF2 show similar trends

**Fig. 4:**
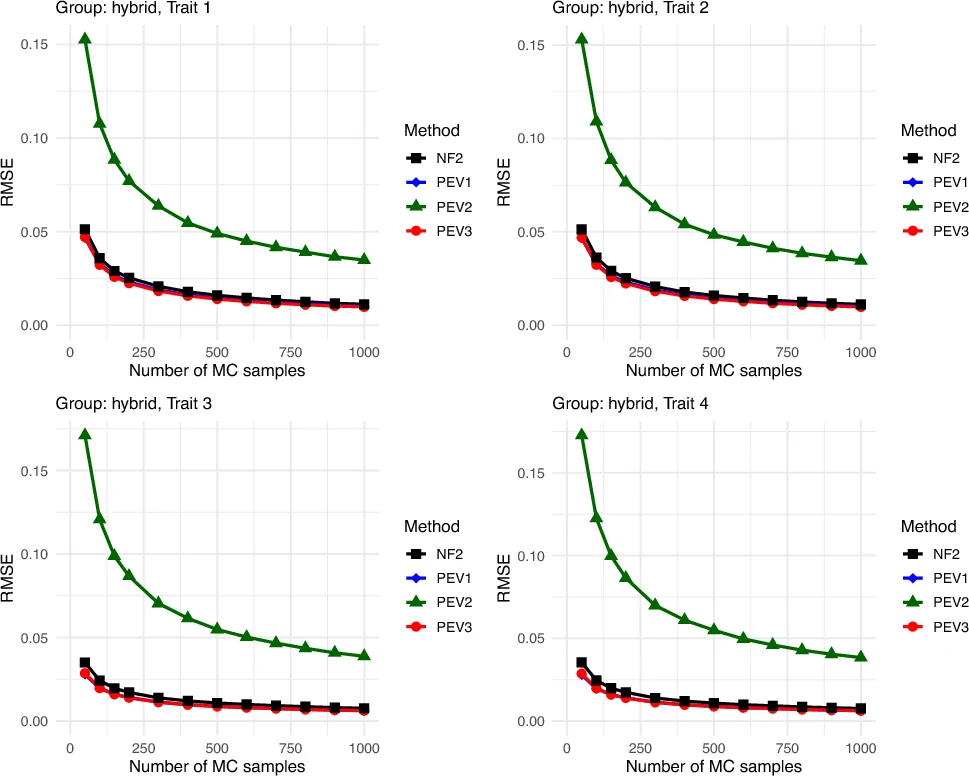
RMSEs of four methods approximating exact reliability using different MC sample sizes (Hybrids). PEV1, PEV3, and NF2 show similar trends

**Fig. 5:**
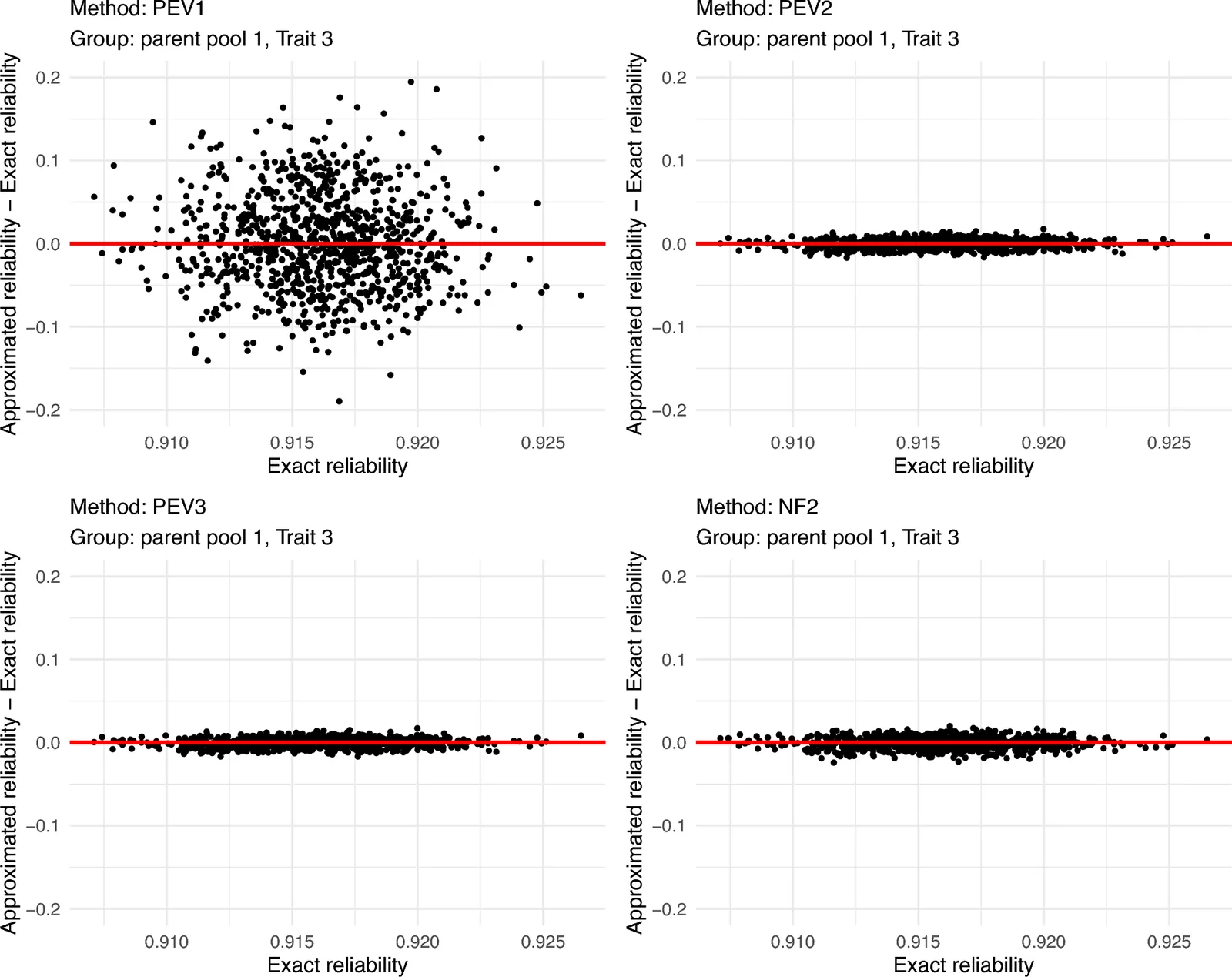
Scatterplots with exact reliability on the x-axis and deviation from exact reliability on the y-axis. Number of MC samples: 500

Figures 2, 3, and 4 show how the RMSEs of the approximation methods decrease as the number of MC samples increases. RMSE of PEV2, PEV3, and NF2 show similar trends for the parent pools 1 and 2 while PEV1 has always higher values. In contrast, for the hybrid group, PEV2 has higher RMSE than the other methods. These methods were unbiased, and as an example, Fig. 5 presents scatterplots, where the unbiasedness of approximation methods can be visually assessed. We selected parent pool 1 as an example, but parent pool 2 and hybrids produce similar figures.

**Fig. 6:**
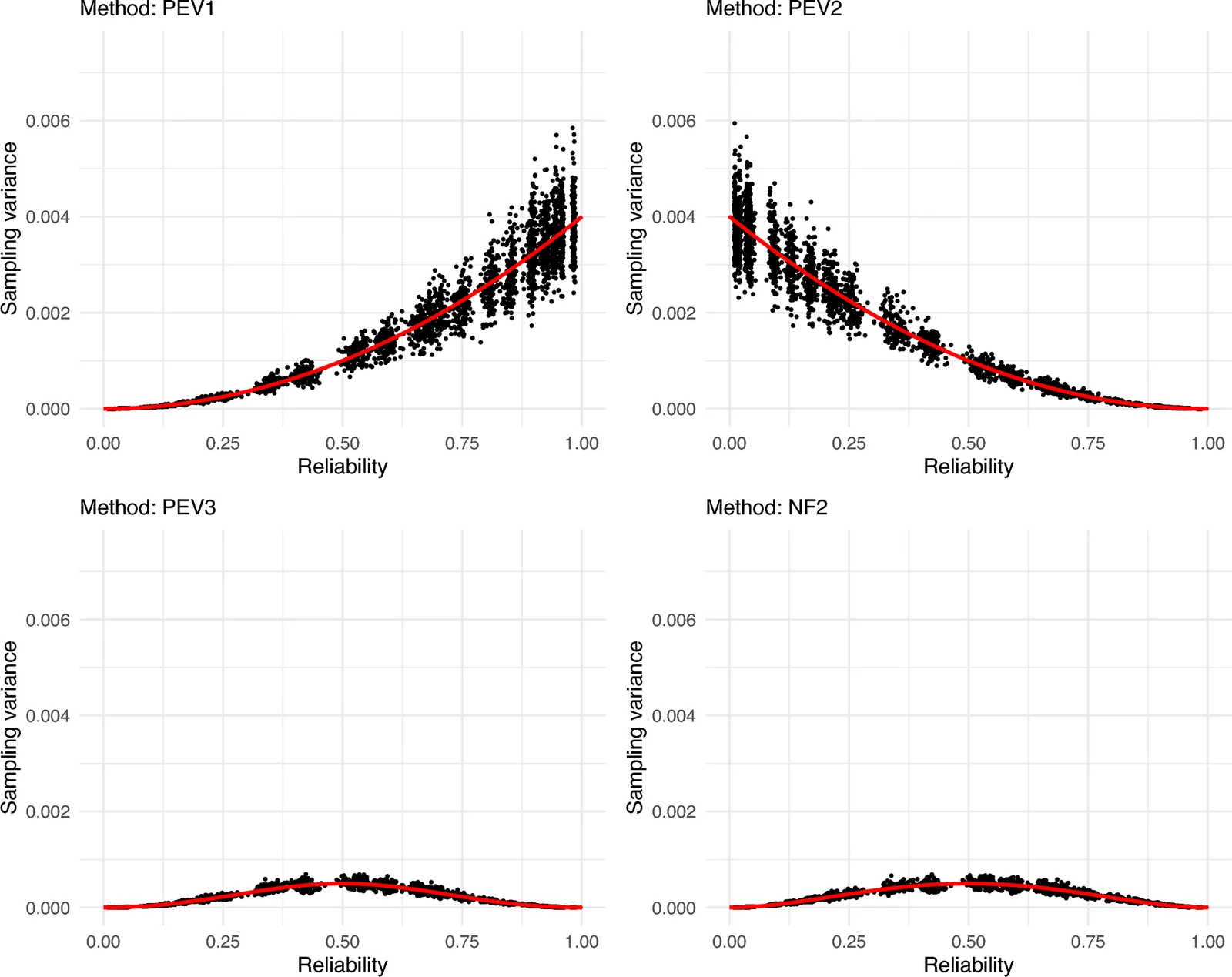
Asymptotic sampling variance (red line) and realized sampling variance (black dots) at different reliability levels

Figure 6 shows how the sampling variances differ across the methods and how the realized sampling variances match the asymptotic sampling variances. It shows that the sampling variance of PEV1 increased as the reliability of the EGVs increased. The sampling variance of PEV2 behaved in the opposite way. The sampling variances of PEV3 and NF2 resembled each other, that is, they had relatively low sampling variance for all reliability values. Thus, differences in performance across reliability levels were explained by sampling variance.

We ran multiple simulations with different sets of parameters to obtain reliabilities across the entire interval (0–1). The realized sampling variances were then combined to create Fig. 6.

**Table 3:** Comparison of different methods for approximating exact reliability and their estimates with different statistics using 500 MC samples

Statistic	Group	Trait	Range of $r^2$	PEV1	PEV2	PEV3	NF2
Correlation	Parent pool 1	1	0.94−0.95	0.046	0.481	0.483	0.399
		2	0.94−0.95	0.050	0.456	0.455	0.368
		3	0.91−0.93	0.004	0.537	0.536	0.418
		4	0.91−0.93	0.035	0.461	0.462	0.370
	Parent pool 2	1	0.94−0.95	−0.001	0.453	0.452	0.328
		2	0.94−0.95	0.052	0.450	0.451	0.364
		3	0.91−0.93	0.081	0.471	0.475	0.401
		4	0.91−0.93	0.082	0.494	0.497	0.414
	Hybrid	1	0.18−0.28	0.717	0.306	0.731	0.686
		2	0.18−0.28	0.717	0.316	0.732	0.690
		3	0.10−0.19	0.778	0.194	0.782	0.711
		4	0.10−0.19	0.779	0.203	0.783	0.716
RMSE	Parent pool 1	1	0.94−0.95	0.057	0.003	0.003	0.004
		2	0.94−0.95	0.057	0.003	0.003	0.005
		3	0.91−0.93	0.056	0.005	0.005	0.007
		4	0.91−0.93	0.056	0.005	0.005	0.007
	Parent pool 2	1	0.94−0.95	0.062	0.003	0.003	0.005
		2	0.94−0.95	0.058	0.003	0.003	0.005
		3	0.91−0.93	0.057	0.005	0.005	0.007
		4	0.91−0.93	0.059	0.005	0.005	0.007
	Hybrid	1	0.18−0.28	0.014	0.049	0.014	0.016
		2	0.18−0.28	0.014	0.048	0.014	0.016
		3	0.10−0.19	0.009	0.055	0.009	0.011
		4	0.10−0.19	0.009	0.055	0.009	0.011
MAX	Parent pool 1	1	0.94−0.95	0.177	0.012	0.011	0.015
		2	0.94−0.95	0.210	0.012	0.013	0.016
		3	0.91−0.93	0.195	0.018	0.017	0.024
		4	0.91−0.93	0.175	0.019	0.019	0.024
	Parent pool 2	1	0.94−0.95	0.208	0.013	0.013	0.016
		2	0.94−0.95	0.196	0.011	0.011	0.017
		3	0.91−0.93	0.190	0.018	0.019	0.022
		4	0.91−0.93	0.223	0.019	0.019	0.024
	Hybrid	1	0.18−0.28	0.054	0.223	0.052	0.059
		2	0.18−0.28	0.057	0.185	0.055	0.070
		3	0.10−0.19	0.045	0.243	0.041	0.063
		4	0.10−0.19	0.044	0.251	0.042	0.053
Intercept	Parent pool 1	1	0.94−0.95	0.946	0.739	0.738	0.808
		2	0.94−0.95	0.945	0.745	0.746	0.820
		3	0.91−0.93	0.916	0.685	0.684	0.764
		4	0.91−0.93	0.914	0.709	0.708	0.781
	Parent pool 2	1	0.94−0.95	0.947	0.757	0.757	0.841
		2	0.94−0.95	0.945	0.754	0.755	0.827
		3	0.91−0.93	0.912	0.700	0.698	0.768
		4	0.91−0.93	0.912	0.701	0.699	0.772
	Hybrid	1	0.18−0.28	0.113	0.211	0.109	0.124
		2	0.18−0.28	0.113	0.210	0.109	0.123
		3	0.10−0.19	0.055	0.134	0.055	0.069
		4	0.10−0.19	0.056	0.134	0.055	0.069
Slope	Parent pool 1	1	0.94−0.95	0.001	0.220	0.221	0.147
		2	0.94−0.95	0.002	0.213	0.213	0.134
		3	0.91−0.93	0.000	0.253	0.254	0.166
		4	0.91−0.93	0.002	0.226	0.227	0.148
	Parent pool 2	1	0.94−0.95	−0.000	0.201	0.201	0.112
		2	0.94−0.95	0.002	0.203	0.203	0.127
		3	0.91−0.93	0.004	0.236	0.239	0.162
		4	0.91−0.93	0.004	0.235	0.237	0.158
	Hybrid	1	0.18−0.28	0.513	0.088	0.529	0.463
		2	0.18−0.28	0.514	0.092	0.530	0.466
		3	0.10−0.19	0.604	0.038	0.609	0.505
		4	0.10−0.19	0.602	0.040	0.607	0.507

The results presented here were obtained using Cholesky MC. To verify that the approximation of the SCA interaction matrix $\textbf{Z}_{\text {cross}}$ does not affect the results, we compared the reliability approximations using Marker-based MC and Cholesky MC to exact reliabilities. As shown in Table 4, the two strategies yielded nearly identical RMSEs across all groups and methods.

Supplementary material 1 provides results for multiple numbers of MC samples and for PEV approximation as well. Supplementary material 2 provides results for a different set of simulation parameters. The results are in agreement with those provided here.

### Computing times

Table 5 presents how the computation time scales with the dataset size and the number of MC samples. For Marker-based MC, computing time increased approximately linearly with both MC sample size and the dataset size. For Cholesky MC, computing time increased close to linearly with the MC sample size but quadratically with dataset size. In our setting, we used $m=50{,}000$ markers, so $n_G<m< 2m$ for all data sets considered, and Cholesky MC therefore requires fewer multiplications than Marker-based MC.

Dataset size refers to the dimension of the MME coefficient matrix, which is affected by the number of individuals and traits. For example, a dataset size of 20,000 can occur when 2000 parents in total have 3000 crosses and four traits are evaluated.

Table 6 presents the computing time required for predicting random effects using the exact method and the PCG algorithm. For relatively small matrices, the exact method was computationally feasible. However, as the dataset size increased, the computing time of the exact method increased rapidly, showing the scalability advantage of the PCG algorithm.

When using any of the MC approximation methods presented, the PCG solver must be applied several times. However, the preconditioner needs to be constructed only once because the coefficient matrix of the MMEs remains the same across the MC iterations. The time needed to construct the preconditioned coefficient matrix was 0.2, 0.7, 2.9, and 15.1 min for dataset sizes of 20,000, 40,000, 80,000, and 160,000, respectively.

## Discussion

We investigated an MC-based method, introduced by García-Cortés et al. (1995) and Hickey et al. (2009), to approximate PEVs and reliabilities of EGVs in an MT-GBLUP framework. We evaluated four previously published PEV approximation formulas, which produced consistent approximations of exact PEVs and reliabilities but with different convergence rates. Although these methods were originally developed for animal models, we applied them here to an MT-GBLUP model. Our findings indicate that both the MC-based approximation method and the PEV formulas are also applicable to genomic models in plant breeding.

Our results are consistent with previous applications of the MC method. Hickey et al. (2009) showed that the methods yield consistent PEV approximations and that realized sampling variances match the asymptotic sampling variances. We also separated the analysis for full-scale and trait-group combinations and noted that metrics such as correlation, intercept, and slope can give overly optimistic results when applied to the full reliability scale. Conversely, they can be overly pessimistic when applied to a narrow range of reliability values. For example, even with a correlation over 0.99, the intercept near 0, and the slope near 1, MAX was 0.223. We argue that both RMSE and MAX should always be reported.

The main strength of this MC-based approximation method is that it yields consistent approximations of exact PEVs and reliabilities and can be easily adapted to many models. Its main drawback is the need to solve the MMEs multiple times. This drawback can be mitigated through parallel computing. Each node can independently handle the MC simulation and solving. With tens of nodes, the required time may be acceptable.

**Table 4:** RMSEs (averaged over 10 MC replicates) between exact and approximated reliabilities using Marker-based MC and Cholesky MC

Group	Method	RMSE (Marker-based MC)	RMSE (Cholesky MC)
Hybrid	PEV1	0.012	0.012
	PEV2	0.052	0.052
	PEV3	0.012	0.012
	NF2	0.014	0.014
Parent pool 1	PEV1	0.059	0.059
	PEV2	0.004	0.004
	PEV3	0.004	0.004
	NF2	0.006	0.006
Parent pool 2	PEV1	0.059	0.058
	PEV2	0.004	0.004
	PEV3	0.004	0.004
	NF2	0.006	0.006

Number of MC samples: 500

**Table 5:** Computing times in seconds for MC sampling of random effects for data sets of different sizes (median of five measurements)

	Marker-based MC				Cholesky MC			
	MC sample size ($n_{\text {mc}}$)				MC sample size ($n_{\text {mc}}$)			
Dataset size	100	500	1000	2000	100	500	1000	2000
20,000	31	138	272	541	2	11	23	46
40,000	68	273	530	1042	7	29	57	114
80,000	157	556	1066	2053	21	71	133	258
160,000	347	1115	2078	4000	51	137	244	461

The maximum dataset size we considered was 160,000. Exact solving took 172.70 min, and solving the MMEs once with PCG took 1.92 min (Table 6). As the MC samples are independent, the MC approximation can be distributed across different computer nodes. If we were to use 50 nodes (each with 10 cores) and 500 MC samples, each node would sample 10 MC samples, and solve the corresponding MMEs 10 times. This means that each node constructs preconditioned coefficient matrix (15.1 min), samples 10 MC samples using Cholesky MC (less than 1 min), and solves the MMEs 10 times using PCG (10×1.92=19.2 minutes). These calculations give an estimate of roughly 35 min of wall-clock time. This means that the approximation method would be over four times faster than the exact method.

The exact time consumption depends on the applied software and algorithms, the number of nodes, and the number of cores; but generally, the approximation method is faster if the number of MC samples stays moderate. For very large datasets, solving the MMEs multiple times may not be possible, and other methods must be used.

The choice between Marker-based MC and Cholesky MC depends on the number of markers and genotyped individuals: Cholesky MC requires fewer computations when $n_G<2m$. Marker-based MC has other advantages. Because genotype matrices contain only three integer values (0, 1, and 2) they can be stored in a compressed form. Freudenberg et al. (2023) developed a fast algorithm for matrix operations involving a genotype matrix. Their results indicate that substantial speed-ups can be achieved for such operations. As another example, Vandenplas et al. (2023) reported that computing times halved when using an algorithm utilizing the compressed form of the genotype matrix.

Not only does the MC-based approximation method circumvent the direct matrix inversion, but it also has the advantage that the MMEs do not have to be stored in the computer’s RAM memory. This is possible because the PCG algorithm supports out-of-core computing, also known as iteration on data, where data are read directly from disk in order to make the required computations in every PCG iteration without forming the MME coefficient matrix in computer RAM (Strandén and Lidauer 1999). For example, storing the 160,000-dimensional coefficient matrix in double precision requires 205 GB of memory, and the space complexity of storing the MT-GBLUP coefficient matrix is $\mathcal {O} (n^2k^2)$, where *n* is the number of random effects and *k* is the number of traits.

We identified some potential limitations in the approximation method. In this study, the same genetic and residual (co)variances were used both to simulate the true data and in the MC simulation. Thus, all VCs were assumed to be known and correctly specified, and no uncertainty in their estimation was considered.

In practice, VCs are unknown and must be estimated from the data. Any misspecification of VCs directly affects the accuracy of PEV and reliability approximations, because they depend on the assumed covariance structure of the model. Therefore, the accuracy of MC-based PEV and reliability approximations is conditional on the quality of the VC estimates. However, assessing the impact of biased VCs on PEV approximation was beyond the scope of this study.

**Table 6:** Computing times in minutes for predicting random effects using the exact method (median of three measurements) and the PCG algorithm (median of five measurements) using datasets of different sizes

Dataset size	Exact method	PCG
20,000	0.37	0.03
40,000	2.70	0.10
80,000	20.53	0.39
160,000	172.70	1.92

Estimation of VCs in large-scale breeding evaluations is computationally demanding. Analytical expectation-maximisation (EM) REML algorithm requires elements of the inverse of the MME coefficient matrix (García-Cortés et al. 1992). These elements correspond to PEVs, and can thus be approximated using the PEV approximation formulas presented in García-Cortés et al. (1995) and Hickey et al. (2009).

García-Cortés et al. (1992) and García-Cortés et al. (1995) presented how MC sampling can be used in the EM-REML algorithm by approximating the inverse elements of the MME coefficient matrix. Later, Matilainen et al. (2012) showed that MC-based EM-REML yields estimates equivalent to those of analytical EM-REML. A recent study by Strandén et al. (2024) presented an augmented AI-REML approach, where augmented MMEs are solved only once per AI-REML iteration, thus improving computational efficiency compared to standard AI-REML.

Because MC-based PEV approximation requires VCs as input, VC estimation must be performed prior to PEV approximation. This adds a substantial computational burden, as VC estimation also requires heavy computation, due to the need to solve the MMEs during each REML iteration. This highlights that PEV and reliability approximation depend highly on reliable and efficient VC estimates, and also on efficient PCG or other iterative solver.

The maximum dataset size in this study was relatively small, and extrapolating computing times to larger datasets should be done with caution. However, because the time complexity of matrix inversion is cubic, while the PCG scales approximately quadratically with the size of the MME coefficient matrix, the relative advantage of the PCG algorithm can be expected to grow with the size of dataset. Our PCG implementation was arguably not the most efficient; software such as MiX99 and BLUPF90 are optimized for solving large MMEs (MiX99 Development Team 2022; Misztal et al. 2014) and would likely perform better. Thus, the reported computing times should be regarded as approximate.

PEVs can also be used to approximate additive genetic variances more accurately. Fernández-González and Sánchez (2025) introduced a method in which PEV is used as a correction factor to improve the accuracy of variance estimation. This highlights that accurate PEVs have applications other than assessing the accuracy of EGVs.

Recent studies have approximated reliabilities of EGVs in genomic evaluations using methods that do not use MC sampling. Ben Zaabza et al. (2022) and Gao et al. (2023) introduced methods approximating reliabilities in single-step GBLUP. Bermann et al. (2022) developed a computationally efficient algorithm to approximate single-step GBLUP reliabilities when using the Algorithm for Proven and Young APY,, (Misztal, 2015). The computational time and memory requirements of their algorithm do not exceed those needed for estimating breeding values. The amount of bias of all these methods is relatively low.

A general limitation of GBLUP models is the need to invert genomic relationship matrices, which makes them infeasible when the number of individuals is very large. An attractive approach is to combine GBLUP with APY, as demonstrated by Bermann et al. (2022). They showed that reliabilities can be approximated in less time than it takes to estimate breeding values. Overall, reliability approximation methods have been studied more extensively in animal breeding than in plant breeding. Even though the same approaches can generally be applied in both fields, their different characteristics should be taken into account, and the applicability of these methods should be studied in plant breeding.

## Conclusion

We investigated MC-based methods for approximating PEVs and reliabilities in the MT-GBLUP model. Our results demonstrated that methods originally developed for animal breeding can also be applied to plant breeding. We showed that these methods yield consistent approximations of the exact PEVs and reliabilities. Our findings are in line with previous studies. However, the need to solve the MMEs multiple times creates a computational burden, which may limit the use of these methods in very large data sets.

## Supplementary Information

Below is the link to the electronic supplementary material.

Supplementary file 1 (pdf 781 KB)

Supplementary file 2 (pdf 834 KB)

## Data Availability

The simulation code to reproduce the analysis is available at Heikkilä et al. (2025).
