# Supplementary material for: Approximating prediction error variances and reliabilities in a multiple-trait genomic prediction model using Monte Carlo sampling
Source: Theor Appl Genet. 2026 Aug 28;139(9):250. doi: 10.1007/s00122-026-05336-0 (PMC13525033; doi:10.1007/s00122-026-05336-0)
Supplement: Supplementary file 1 [file 122_2026_5336_MOESM1_ESM.pdf]

Supplementary material 1 for:

Approximating prediction error variances and reliabilities in multiple-trait genomic prediction model using Monte Carlo sampling

Antero Heikkilä[1*], Ismo Strandén[2], Martin Lidauer[2], Klaus Nordhausen[3], Sara Taskinen[1]

[1*]Department of Mathematics and Statistics, University of Jyväskylä, Jyväskylä, FI-40014, Finland.
[2] Natural Resources Institute Finland (Luke), Jokioinen, FI-31600, Finland.
[3]Department of Mathematics and Statistics, University of Helsinki, Helsinki, FI-00014, Finland.

*Corresponding author(s). E-mail(s): antero.j.heikkila@jyu.fi;
Contributing authors: ismo.stranden@luke.fi ; martin.lidauer@luke.fi ; klaus.nordhausen@helsinki.fi; sara.taskinen@jyu.fi;

This supplementary material contains additional tables and figures related to the original setting described in the article. We performed the analysis using three MC sample sizes: 100, 500, and 1,000. Results related to reliability approximation are presented in Tables S1.1–S1.3 and S1.7–S1.9, and in Figures S1.1-S1.3. Results related to PEV approximation are presented in Tables S1.4–S1.6 and S1.10–S1.12, and in Figures S1.4–S1.6.

Table S1.1: Comparison of different methods approximating the exact reliability and their estimates with different statistics using 100 MC samples

Statistic	PEV1	PEV2	PEV3	NF2
Correlation	0.978	0.936	0.996	0.995
RMSE	0.060	0.105	0.025	0.029
MAX	0.562	0.624	0.147	0.185
Intercept	0.014	0.036	0.001	0.000
Slope	0.957	0.879	0.994	0.994

Table S1.2: Comparison of different methods approximating the exact reliability and their estimates with different statistics using 500 MC samples

Statistic	PEV1	PEV2	PEV3	NF2
Correlation	0.996	0.986	0.999	0.999
RMSE	0.026	0.047	0.011	0.013
MAX	0.223	0.251	0.055	0.070
Intercept	0.002	0.008	0.000	0.000
Slope	0.993	0.973	0.999	0.999

Table S1.3: Comparison of different methods approximating the exact reliability and their estimates with different statistics using 1,000 MC samples

Statistic	PEV1	PEV2	PEV3	NF2
Correlation	0.998	0.993	1.000	1.000
RMSE	0.018	0.034	0.008	0.009
MAX	0.166	0.163	0.044	0.044
Intercept	0.001	0.004	0.000	0.000
Slope	0.996	0.986	0.999	0.999

Table S1.4: Comparison of different methods approximating the exact PEV and their estimates with different statistics using 100 MC samples

Statistic	PEV1	PEV2	PEV3	NF2
Correlation	0.185	0.637	0.891	0.829
RMSE	0.054	0.011	0.005	0.006
MAX	0.563	0.062	0.049	0.079
Intercept	0.077	0.047	0.016	0.024
Slope	0.031	0.405	0.794	0.696

Table S1.5: Comparison of different methods approximating the exact PEV and their estimates with different statistics using 500 MC samples

Statistic	PEV1	PEV2	PEV3	NF2
Correlation	0.338	0.878	0.976	0.960
RMSE	0.024	0.005	0.002	0.003
MAX	0.221	0.025	0.019	0.024
Intercept	0.069	0.018	0.004	0.006
Slope	0.125	0.769	0.951	0.929

Table S1.6: Comparison of different methods approximating the exact PEV and their estimates with different statistics using 1,000 MC samples

Statistic	PEV1	PEV2	PEV3	NF2
Correlation	0.479	0.933	0.988	0.980
RMSE	0.017	0.004	0.001	0.002
MAX	0.166	0.017	0.014	0.020
Intercept	0.061	0.010	0.002	0.003
Slope	0.233	0.871	0.975	0.962

Table S1.7: Comparison of different methods for approximating exact reliability and their estimates with different statistics using 100 MC samples

**Statistic**	**Group**	**Trait**	**Range of r^2**	**PEV1**	**PEV2**	**PEV3**	**NF2**
Correlation	parent pool 1	1	0.94-0.95	0.086	0.244	0.248	0.233
		2	0.94-0.95	0.052	0.253	0.253	0.205
		3	0.91-0.93	-0.055	0.242	0.235	0.131
		4	0.91-0.93	-0.069	0.228	0.220	0.113
	parent pool 2	1	0.94-0.95	-0.046	0.174	0.172	0.091
		2	0.94-0.95	0.006	0.213	0.214	0.158
		3	0.91-0.93	0.057	0.237	0.241	0.199
		4	0.91-0.93	0.104	0.239	0.248	0.244
	hybrid	1	0.18-0.28	0.433	0.136	0.442	0.398
		2	0.18-0.28	0.435	0.138	0.445	0.400
		3	0.10-0.19	0.502	0.078	0.504	0.421
		4	0.10-0.19	0.512	0.087	0.514	0.429
RMSE	parent pool 1	1	0.94-0.95	0.133	0.007	0.007	0.010
		2	0.94-0.95	0.132	0.008	0.008	0.011
		3	0.91-0.93	0.138	0.012	0.012	0.016
		4	0.91-0.93	0.135	0.012	0.012	0.016
	parent pool 2	1	0.94-0.95	0.132	0.007	0.007	0.010
		2	0.94-0.95	0.129	0.008	0.008	0.010
		3	0.91-0.93	0.133	0.011	0.011	0.016
		4	0.91-0.93	0.129	0.012	0.012	0.015
	hybrid	1	0.18-0.28	0.033	0.108	0.032	0.036
		2	0.18-0.28	0.033	0.109	0.032	0.036
		3	0.10-0.19	0.020	0.121	0.020	0.024
		4	0.10-0.19	0.020	0.123	0.020	0.025
MAX	parent pool 1	1	0.94-0.95	0.438	0.029	0.032	0.048
		2	0.94-0.95	0.442	0.030	0.029	0.043
		3	0.91-0.93	0.467	0.043	0.041	0.081
		4	0.91-0.93	0.436	0.047	0.046	0.052
	parent pool 2	1	0.94-0.95	0.520	0.028	0.031	0.056
		2	0.94-0.95	0.519	0.029	0.030	0.041
		3	0.91-0.93	0.562	0.035	0.034	0.053
		4	0.91-0.93	0.517	0.050	0.048	0.058
	hybrid	1	0.18-0.28	0.169	0.424	0.146	0.153
		2	0.18-0.28	0.146	0.430	0.147	0.185
		3	0.10-0.19	0.087	0.491	0.089	0.110
		4	0.10-0.19	0.089	0.624	0.084	0.114
Intercept	parent pool 1	1	0.94-0.95	0.946	0.895	0.894	0.911
		2	0.94-0.95	0.946	0.894	0.894	0.916
		3	0.91-0.93	0.917	0.864	0.866	0.895
		4	0.91-0.93	0.917	0.865	0.868	0.898
	parent pool 2	1	0.94-0.95	0.948	0.910	0.911	0.933
		2	0.94-0.95	0.947	0.903	0.903	0.922
		3	0.91-0.93	0.915	0.860	0.859	0.882
		4	0.91-0.93	0.914	0.864	0.862	0.874
	hybrid	1	0.18-0.28	0.191	0.227	0.189	0.196
		2	0.18-0.28	0.190	0.227	0.188	0.196
		3	0.10-0.19	0.106	0.139	0.106	0.115
		4	0.10-0.19	0.105	0.138	0.105	0.115
Slope	parent pool 1	1	0.94-0.95	0.001	0.055	0.056	0.038
		2	0.94-0.95	0.001	0.056	0.056	0.033
		3	0.91-0.93	-0.001	0.057	0.055	0.023
		4	0.91-0.93	-0.001	0.056	0.053	0.020
	parent pool 2	1	0.94-0.95	-0.001	0.039	0.038	0.015
		2	0.94-0.95	0.000	0.046	0.046	0.026
		3	0.91-0.93	0.001	0.062	0.062	0.037
		4	0.91-0.93	0.002	0.057	0.059	0.046
	hybrid	1	0.18-0.28	0.176	0.019	0.182	0.151
		2	0.18-0.28	0.178	0.019	0.184	0.151
		3	0.10-0.19	0.240	0.007	0.241	0.172
		4	0.10-0.19	0.246	0.008	0.246	0.174

Table S1.8: Comparison of different methods for approximating exact reliability and their estimates with different statistics using 500 MC samples

**Statistic**	**Group**	**Trait**	**Range of r^2**	**PEV1**	**PEV2**	**PEV3**	**NF2**
Correlation	parent pool 1	1	0.94-0.95	0.046	0.481	0.483	0.399
		2	0.94-0.95	0.050	0.456	0.455	0.368
		3	0.91-0.93	0.004	0.537	0.536	0.418
		4	0.91-0.93	0.035	0.461	0.462	0.370
	parent pool 2	1	0.94-0.95	-0.001	0.453	0.452	0.328
		2	0.94-0.95	0.052	0.450	0.451	0.364
		3	0.91-0.93	0.081	0.471	0.475	0.401
		4	0.91-0.93	0.082	0.494	0.497	0.414
	hybrid	1	0.18-0.28	0.717	0.306	0.731	0.686
		2	0.18-0.28	0.717	0.316	0.732	0.690
		3	0.10-0.19	0.778	0.194	0.782	0.711
		4	0.10-0.19	0.779	0.203	0.783	0.716
RMSE	parent pool 1	1	0.94-0.95	0.057	0.003	0.003	0.004
		2	0.94-0.95	0.057	0.003	0.003	0.005
		3	0.91-0.93	0.056	0.005	0.005	0.007
		4	0.91-0.93	0.056	0.005	0.005	0.007
	parent pool 2	1	0.94-0.95	0.062	0.003	0.003	0.005
		2	0.94-0.95	0.058	0.003	0.003	0.005
		3	0.91-0.93	0.057	0.005	0.005	0.007
		4	0.91-0.93	0.059	0.005	0.005	0.007
	hybrid	1	0.18-0.28	0.014	0.049	0.014	0.016
		2	0.18-0.28	0.014	0.048	0.014	0.016
		3	0.10-0.19	0.009	0.055	0.009	0.011
		4	0.10-0.19	0.009	0.055	0.009	0.011
MAX	parent pool 1	1	0.94-0.95	0.177	0.012	0.011	0.015
		2	0.94-0.95	0.210	0.012	0.013	0.016
		3	0.91-0.93	0.195	0.018	0.017	0.024
		4	0.91-0.93	0.175	0.019	0.019	0.024
	parent pool 2	1	0.94-0.95	0.208	0.013	0.013	0.016
		2	0.94-0.95	0.196	0.011	0.011	0.017
		3	0.91-0.93	0.190	0.018	0.019	0.022
		4	0.91-0.93	0.223	0.019	0.019	0.024
	hybrid	1	0.18-0.28	0.054	0.223	0.052	0.059
		2	0.18-0.28	0.057	0.185	0.055	0.070
		3	0.10-0.19	0.045	0.243	0.041	0.063
		4	0.10-0.19	0.044	0.251	0.042	0.053
Intercept	parent pool 1	1	0.94-0.95	0.946	0.739	0.738	0.808
		2	0.94-0.95	0.945	0.745	0.746	0.820
		3	0.91-0.93	0.916	0.685	0.684	0.764
		4	0.91-0.93	0.914	0.709	0.708	0.781
	parent pool 2	1	0.94-0.95	0.947	0.757	0.757	0.841
		2	0.94-0.95	0.945	0.754	0.755	0.827
		3	0.91-0.93	0.912	0.700	0.698	0.768
		4	0.91-0.93	0.912	0.701	0.699	0.772
	hybrid	1	0.18-0.28	0.113	0.211	0.109	0.124
		2	0.18-0.28	0.113	0.210	0.109	0.123
		3	0.10-0.19	0.055	0.134	0.055	0.069
		4	0.10-0.19	0.056	0.134	0.055	0.069
Slope	parent pool 1	1	0.94-0.95	0.001	0.220	0.221	0.147
		2	0.94-0.95	0.002	0.213	0.213	0.134
		3	0.91-0.93	0.000	0.253	0.254	0.166
		4	0.91-0.93	0.002	0.226	0.227	0.148
	parent pool 2	1	0.94-0.95	-0.000	0.201	0.201	0.112
		2	0.94-0.95	0.002	0.203	0.203	0.127
		3	0.91-0.93	0.004	0.236	0.239	0.162
		4	0.91-0.93	0.004	0.235	0.237	0.158
	hybrid	1	0.18-0.28	0.513	0.088	0.529	0.463
		2	0.18-0.28	0.514	0.092	0.530	0.466
		3	0.10-0.19	0.604	0.038	0.609	0.505
		4	0.10-0.19	0.602	0.040	0.607	0.507

Table S1.9: Comparison of different methods for approximating exact reliability and their estimates with different statistics using 1,000 MC samples

Statistic	Group	Trait	Range of r^2	PEV1	PEV2	PEV3	NF2
Correlation	parent pool 1	1	0.94-0.95	0.028	0.605	0.605	0.483
		2	0.94-0.95	0.075	0.589	0.591	0.499
		3	0.91-0.93	0.024	0.637	0.636	0.512
		4	0.91-0.93	0.072	0.605	0.608	0.508
	parent pool 2	1	0.94-0.95	0.099	0.572	0.573	0.482
		2	0.94-0.95	0.101	0.603	0.605	0.516
		3	0.91-0.93	0.124	0.598	0.603	0.529
		4	0.91-0.93	0.117	0.624	0.627	0.551
	hybrid	1	0.18-0.28	0.823	0.392	0.834	0.797
		2	0.18-0.28	0.825	0.403	0.837	0.802
		3	0.10-0.19	0.867	0.269	0.869	0.818
		4	0.10-0.19	0.867	0.271	0.870	0.822
RMSE	parent pool 1	1	0.94-0.95	0.042	0.002	0.002	0.003
		2	0.94-0.95	0.042	0.002	0.002	0.003
		3	0.91-0.93	0.039	0.004	0.004	0.005
		4	0.91-0.93	0.042	0.004	0.004	0.005
	parent pool 2	1	0.94-0.95	0.041	0.002	0.002	0.003
		2	0.94-0.95	0.041	0.002	0.002	0.003
		3	0.91-0.93	0.040	0.004	0.004	0.005
		4	0.91-0.93	0.040	0.004	0.004	0.005
	hybrid	1	0.18-0.28	0.010	0.035	0.010	0.011
		2	0.18-0.28	0.010	0.035	0.010	0.011
		3	0.10-0.19	0.006	0.039	0.006	0.008
		4	0.10-0.19	0.006	0.038	0.006	0.008
MAX	parent pool 1	1	0.94-0.95	0.151	0.008	0.008	0.012
		2	0.94-0.95	0.163	0.008	0.009	0.014
		3	0.91-0.93	0.143	0.013	0.013	0.018
		4	0.91-0.93	0.166	0.012	0.012	0.020
	parent pool 2	1	0.94-0.95	0.139	0.008	0.008	0.011
		2	0.94-0.95	0.128	0.007	0.007	0.009
		3	0.91-0.93	0.124	0.014	0.014	0.014
		4	0.91-0.93	0.139	0.014	0.014	0.017
	hybrid	1	0.18-0.28	0.044	0.148	0.039	0.044
		2	0.18-0.28	0.047	0.152	0.044	0.042
		3	0.10-0.19	0.028	0.163	0.025	0.035
		4	0.10-0.19	0.024	0.154	0.024	0.032
Intercept	parent pool 1	1	0.94-0.95	0.946	0.614	0.614	0.731
		2	0.94-0.95	0.944	0.612	0.611	0.718
		3	0.91-0.93	0.915	0.552	0.551	0.668
		4	0.91-0.93	0.911	0.569	0.567	0.675
	parent pool 2	1	0.94-0.95	0.943	0.633	0.633	0.741
		2	0.94-0.95	0.943	0.610	0.610	0.719
		3	0.91-0.93	0.908	0.563	0.560	0.659
		4	0.91-0.93	0.908	0.572	0.570	0.658
	hybrid	1	0.18-0.28	0.074	0.196	0.070	0.084
		2	0.18-0.28	0.074	0.195	0.070	0.084
		3	0.10-0.19	0.035	0.129	0.034	0.046
		4	0.10-0.19	0.035	0.129	0.034	0.045
Slope	parent pool 1	1	0.94-0.95	0.001	0.352	0.352	0.229
		2	0.94-0.95	0.003	0.353	0.354	0.242
		3	0.91-0.93	0.002	0.398	0.398	0.271
		4	0.91-0.93	0.005	0.379	0.381	0.263
	parent pool 2	1	0.94-0.95	0.004	0.332	0.331	0.218
		2	0.94-0.95	0.004	0.356	0.356	0.241
		3	0.91-0.93	0.009	0.386	0.389	0.281
		4	0.91-0.93	0.009	0.375	0.378	0.282
	hybrid	1	0.18-0.28	0.679	0.153	0.697	0.637
		2	0.18-0.28	0.679	0.159	0.697	0.638
		3	0.10-0.19	0.751	0.073	0.755	0.670
		4	0.10-0.19	0.750	0.075	0.755	0.675

Table S1.10: Comparison of different methods for approximating exact PEV and their estimates with different statistics using 100 MC samples

Statistic	Group	Trait	Range of PEV	PEV1	PEV2	PEV3	NF2
Correlation	parent pool 1	1	0.05-0.06	0.043	0.228	0.231	0.199
		2	0.05-0.06	0.033	0.261	0.261	0.202
		3	0.07-0.10	-0.073	0.261	0.253	0.134
		4	0.07-0.09	-0.101	0.242	0.232	0.105
	parent pool 2	1	0.05-0.06	-0.101	0.201	0.195	0.072
		2	0.05-0.06	0.017	0.239	0.238	0.180
		3	0.07-0.10	0.019	0.263	0.264	0.194
		4	0.07-0.10	0.116	0.265	0.273	0.268
	hybrid	1	0.07-0.09	0.600	0.209	0.607	0.561
		2	0.07-0.09	0.600	0.212	0.606	0.559
		3	0.08-0.10	0.776	0.203	0.777	0.709
		4	0.08-0.10	0.781	0.192	0.779	0.706
RMSE	parent pool 1	1	0.05-0.06	0.133	0.007	0.007	0.010
		2	0.05-0.06	0.132	0.008	0.008	0.011
		3	0.07-0.10	0.138	0.012	0.012	0.016
		4	0.07-0.09	0.135	0.012	0.012	0.016
	parent pool 2	1	0.05-0.06	0.133	0.007	0.007	0.010
		2	0.05-0.06	0.129	0.008	0.008	0.010
		3	0.07-0.10	0.133	0.011	0.011	0.016
		4	0.07-0.10	0.129	0.012	0.012	0.015
	hybrid	1	0.07-0.09	0.003	0.011	0.003	0.004
		2	0.07-0.09	0.003	0.011	0.003	0.004
		3	0.08-0.10	0.002	0.012	0.002	0.002
		4	0.08-0.10	0.002	0.012	0.002	0.002
MAX	parent pool 1	1	0.05-0.06	0.450	0.029	0.032	0.049
		2	0.05-0.06	0.441	0.030	0.029	0.043
		3	0.07-0.10	0.478	0.043	0.041	0.079
		4	0.07-0.09	0.434	0.047	0.046	0.052
	parent pool 2	1	0.05-0.06	0.520	0.028	0.031	0.057
		2	0.05-0.06	0.510	0.029	0.030	0.041
		3	0.07-0.10	0.563	0.036	0.035	0.053
		4	0.07-0.10	0.511	0.051	0.049	0.058
	hybrid	1	0.07-0.09	0.017	0.043	0.014	0.015
		2	0.07-0.09	0.015	0.044	0.014	0.018
		3	0.08-0.10	0.009	0.051	0.009	0.012
		4	0.08-0.10	0.009	0.062	0.008	0.011
Intercept	parent pool 1	1	0.05-0.06	0.053	0.050	0.050	0.051
		2	0.05-0.06	0.053	0.050	0.050	0.051
		3	0.07-0.10	0.084	0.078	0.079	0.082
		4	0.07-0.09	0.084	0.079	0.079	0.082
	parent pool 2	1	0.05-0.06	0.053	0.051	0.051	0.052
		2	0.05-0.06	0.053	0.050	0.050	0.051
		3	0.07-0.10	0.084	0.078	0.078	0.080
		4	0.07-0.10	0.084	0.078	0.078	0.079
	hybrid	1	0.07-0.09	0.050	0.073	0.050	0.053
		2	0.07-0.09	0.050	0.073	0.050	0.054
		3	0.08-0.10	0.035	0.083	0.035	0.044
		4	0.08-0.10	0.035	0.083	0.035	0.044
Slope	parent pool 1	1	0.05-0.06	0.001	0.054	0.054	0.034
		2	0.05-0.06	0.000	0.060	0.060	0.034
		3	0.07-0.10	-0.002	0.064	0.062	0.025
		4	0.07-0.09	-0.002	0.062	0.059	0.020
	parent pool 2	1	0.05-0.06	-0.001	0.048	0.047	0.013
		2	0.05-0.06	0.000	0.054	0.054	0.031
		3	0.07-0.10	0.000	0.073	0.072	0.039
		4	0.07-0.10	0.003	0.068	0.070	0.054
	hybrid	1	0.07-0.09	0.346	0.045	0.355	0.308
		2	0.07-0.09	0.347	0.045	0.354	0.305
		3	0.08-0.10	0.592	0.039	0.592	0.493
		4	0.08-0.10	0.595	0.037	0.592	0.488

Table S1.11: Comparison of different methods for approximating exact PEV and their estimates with different statistics using 500 MC samples

Statistic	Group	Trait	Range of PEV	PEV1	PEV2	PEV3	NF2
Correlation	parent pool 1	1	0.05-0.06	0.055	0.475	0.477	0.400
		2	0.05-0.06	0.055	0.470	0.470	0.383
		3	0.07-0.10	0.022	0.552	0.552	0.442
		4	0.07-0.09	0.035	0.499	0.500	0.402
	parent pool 2	1	0.05-0.06	-0.009	0.463	0.461	0.330
		2	0.05-0.06	0.093	0.467	0.469	0.398
		3	0.07-0.10	0.074	0.502	0.506	0.422
		4	0.07-0.10	0.073	0.517	0.519	0.428
	hybrid	1	0.07-0.09	0.854	0.437	0.861	0.829
		2	0.07-0.09	0.854	0.451	0.863	0.832
		3	0.08-0.10	0.938	0.410	0.939	0.912
		4	0.08-0.10	0.937	0.402	0.939	0.912
RMSE	parent pool 1	1	0.05-0.06	0.057	0.003	0.003	0.004
		2	0.05-0.06	0.057	0.003	0.003	0.005
		3	0.07-0.10	0.056	0.005	0.005	0.007
		4	0.07-0.09	0.056	0.005	0.005	0.007
	parent pool 2	1	0.05-0.06	0.062	0.003	0.003	0.005
		2	0.05-0.06	0.058	0.003	0.003	0.005
		3	0.07-0.10	0.057	0.005	0.005	0.007
		4	0.07-0.10	0.059	0.005	0.005	0.007
	hybrid	1	0.07-0.09	0.001	0.005	0.001	0.002
		2	0.07-0.09	0.001	0.005	0.001	0.002
		3	0.08-0.10	0.001	0.005	0.001	0.001
		4	0.08-0.10	0.001	0.005	0.001	0.001
MAX	parent pool 1	1	0.05-0.06	0.177	0.012	0.011	0.015
		2	0.05-0.06	0.213	0.012	0.013	0.016
		3	0.07-0.10	0.192	0.018	0.017	0.024
		4	0.07-0.09	0.180	0.019	0.019	0.024
	parent pool 2	1	0.05-0.06	0.206	0.013	0.013	0.016
		2	0.05-0.06	0.198	0.011	0.011	0.017
		3	0.07-0.10	0.190	0.018	0.018	0.021
		4	0.07-0.10	0.221	0.019	0.019	0.024
	hybrid	1	0.07-0.09	0.005	0.022	0.005	0.006
		2	0.07-0.09	0.006	0.019	0.006	0.007
		3	0.08-0.10	0.004	0.024	0.004	0.006
		4	0.08-0.10	0.004	0.025	0.004	0.005
Intercept	parent pool 1	1	0.05-0.06	0.053	0.041	0.041	0.045
		2	0.05-0.06	0.053	0.041	0.041	0.045
		3	0.07-0.10	0.084	0.061	0.061	0.069
		4	0.07-0.09	0.084	0.063	0.063	0.070
	parent pool 2	1	0.05-0.06	0.053	0.041	0.041	0.047
		2	0.05-0.06	0.053	0.041	0.041	0.045
		3	0.07-0.10	0.083	0.062	0.061	0.069
		4	0.07-0.10	0.083	0.062	0.062	0.069
	hybrid	1	0.07-0.09	0.021	0.062	0.020	0.024
		2	0.07-0.09	0.021	0.062	0.020	0.024
		3	0.08-0.10	0.011	0.072	0.010	0.015
		4	0.08-0.10	0.011	0.072	0.010	0.015
Slope	parent pool 1	1	0.05-0.06	0.002	0.225	0.227	0.152
		2	0.05-0.06	0.002	0.226	0.226	0.144
		3	0.07-0.10	0.001	0.270	0.272	0.182
		4	0.07-0.09	0.002	0.253	0.254	0.167
	parent pool 2	1	0.05-0.06	-0.000	0.220	0.219	0.122
		2	0.05-0.06	0.003	0.221	0.221	0.145
		3	0.07-0.10	0.004	0.263	0.266	0.180
		4	0.07-0.10	0.004	0.262	0.264	0.175
	hybrid	1	0.07-0.09	0.731	0.190	0.743	0.687
		2	0.07-0.09	0.729	0.197	0.741	0.688
		3	0.08-0.10	0.878	0.163	0.879	0.826
		4	0.08-0.10	0.876	0.161	0.878	0.828

Table S1.12: Comparison of different methods for approximating exact PEV and their estimates with different statistics using 1,000 MC samples

Statistic	Group	Trait	Range of PEV	PEV1	PEV2	PEV3	NF2
Correlation	parent pool 1	1	0.05-0.06	0.045	0.598	0.598	0.485
		2	0.05-0.06	0.071	0.602	0.604	0.508
		3	0.07-0.10	0.037	0.655	0.655	0.535
		4	0.07-0.09	0.066	0.638	0.641	0.535
	parent pool 2	1	0.05-0.06	0.116	0.584	0.586	0.500
		2	0.05-0.06	0.131	0.623	0.625	0.546
		3	0.07-0.10	0.130	0.635	0.639	0.563
		4	0.07-0.10	0.125	0.653	0.657	0.582
	hybrid	1	0.07-0.09	0.919	0.555	0.924	0.903
		2	0.07-0.09	0.919	0.565	0.925	0.905
		3	0.08-0.10	0.967	0.526	0.968	0.952
		4	0.08-0.10	0.967	0.529	0.968	0.953
RMSE	parent pool 1	1	0.05-0.06	0.042	0.002	0.002	0.003
		2	0.05-0.06	0.043	0.002	0.002	0.003
		3	0.07-0.10	0.039	0.004	0.004	0.005
		4	0.07-0.09	0.042	0.004	0.004	0.005
	parent pool 2	1	0.05-0.06	0.041	0.002	0.002	0.003
		2	0.05-0.06	0.041	0.002	0.002	0.003
		3	0.07-0.10	0.040	0.004	0.004	0.005
		4	0.07-0.10	0.040	0.004	0.004	0.005
	hybrid	1	0.07-0.09	0.001	0.003	0.001	0.001
		2	0.07-0.09	0.001	0.003	0.001	0.001
		3	0.08-0.10	0.001	0.004	0.001	0.001
		4	0.08-0.10	0.001	0.004	0.001	0.001
MAX	parent pool 1	1	0.05-0.06	0.152	0.008	0.008	0.012
		2	0.05-0.06	0.166	0.008	0.009	0.014
		3	0.07-0.10	0.143	0.013	0.013	0.017
		4	0.07-0.09	0.166	0.012	0.012	0.020
	parent pool 2	1	0.05-0.06	0.140	0.008	0.008	0.011
		2	0.05-0.06	0.131	0.007	0.007	0.009
		3	0.07-0.10	0.121	0.014	0.014	0.014
		4	0.07-0.10	0.137	0.014	0.014	0.017
	hybrid	1	0.07-0.09	0.005	0.015	0.004	0.004
		2	0.07-0.09	0.005	0.016	0.005	0.004
		3	0.08-0.10	0.003	0.017	0.003	0.004
		4	0.08-0.10	0.002	0.016	0.003	0.003
Intercept	parent pool 1	1	0.05-0.06	0.053	0.034	0.034	0.040
		2	0.05-0.06	0.053	0.033	0.033	0.040
		3	0.07-0.10	0.084	0.048	0.048	0.059
		4	0.07-0.09	0.083	0.050	0.049	0.060
	parent pool 2	1	0.05-0.06	0.053	0.034	0.034	0.040
		2	0.05-0.06	0.053	0.033	0.033	0.039
		3	0.07-0.10	0.083	0.048	0.048	0.058
		4	0.07-0.10	0.083	0.049	0.049	0.058
	hybrid	1	0.07-0.09	0.012	0.053	0.011	0.014
		2	0.07-0.09	0.012	0.052	0.011	0.014
		3	0.08-0.10	0.006	0.062	0.006	0.008
		4	0.08-0.10	0.006	0.062	0.005	0.008
Slope	parent pool 1	1	0.05-0.06	0.002	0.362	0.363	0.237
		2	0.05-0.06	0.003	0.371	0.372	0.254
		3	0.07-0.10	0.003	0.423	0.424	0.294
		4	0.07-0.09	0.005	0.410	0.412	0.288
	parent pool 2	1	0.05-0.06	0.005	0.362	0.361	0.240
		2	0.05-0.06	0.006	0.381	0.381	0.264
		3	0.07-0.10	0.010	0.421	0.425	0.311
		4	0.07-0.10	0.010	0.411	0.414	0.313
	hybrid	1	0.07-0.09	0.844	0.314	0.856	0.820
		2	0.07-0.09	0.842	0.320	0.853	0.818
		3	0.08-0.10	0.934	0.275	0.935	0.905
		4	0.08-0.10	0.935	0.280	0.936	0.908

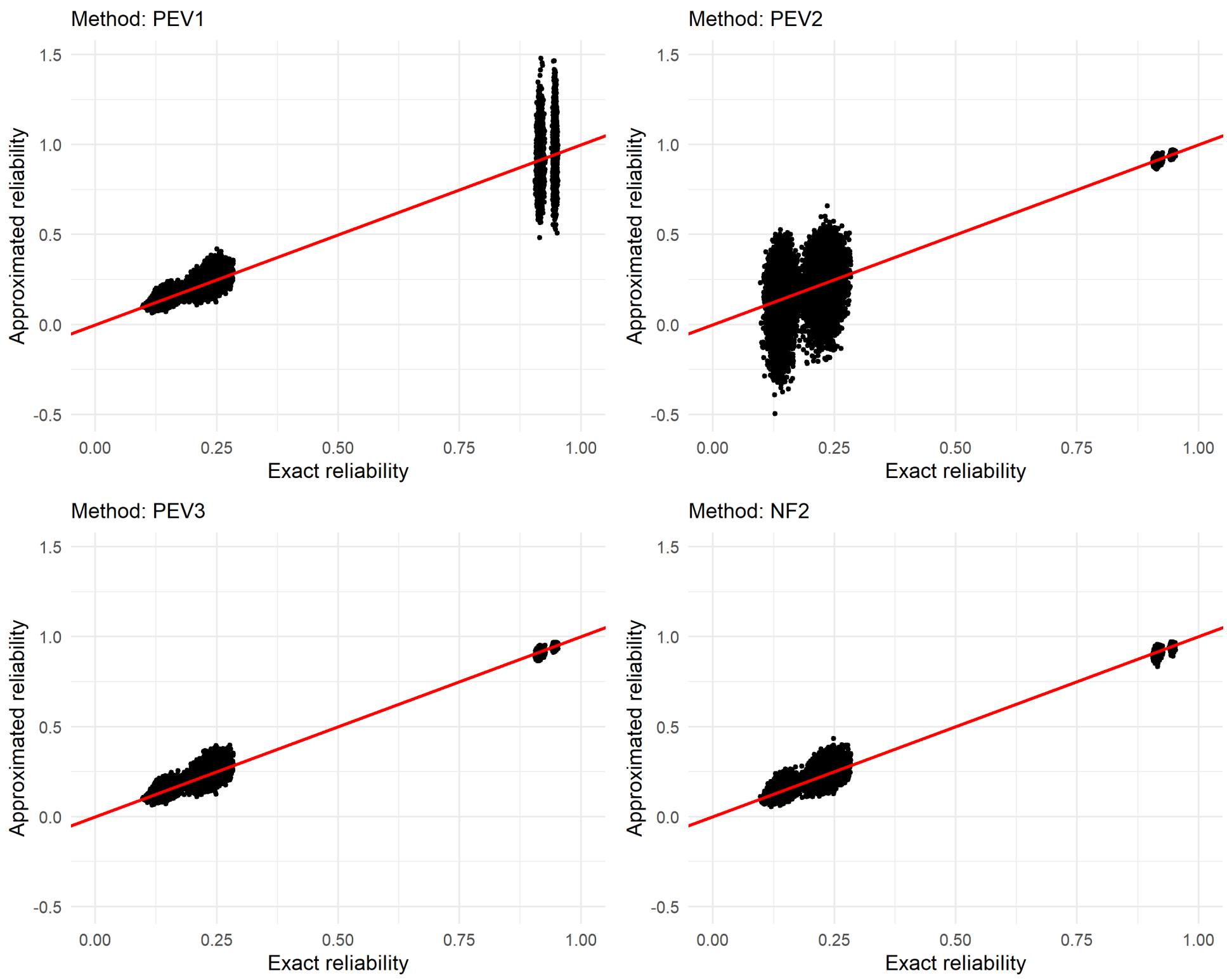

Fig. S1.1: Scatterplots with exact reliability on the x-axis and approximated reliability on the y-axis. Number of MC samples: 100. The red line represents $y = x$

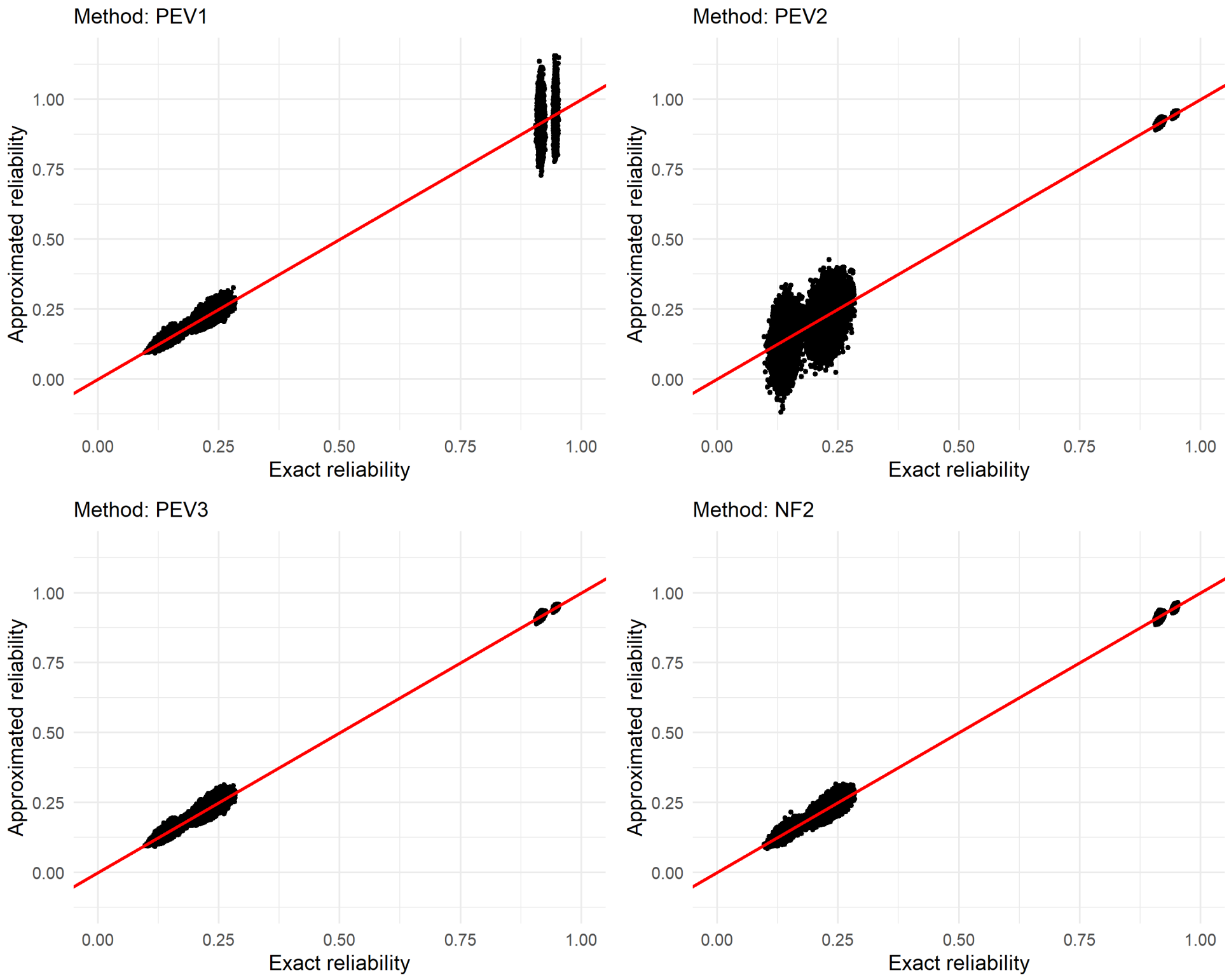

Fig. S1.2: Scatterplots with exact reliability on the x-axis and approximated reliability on the y-axis. Number of MC samples: 500. The red line represents $y = x$

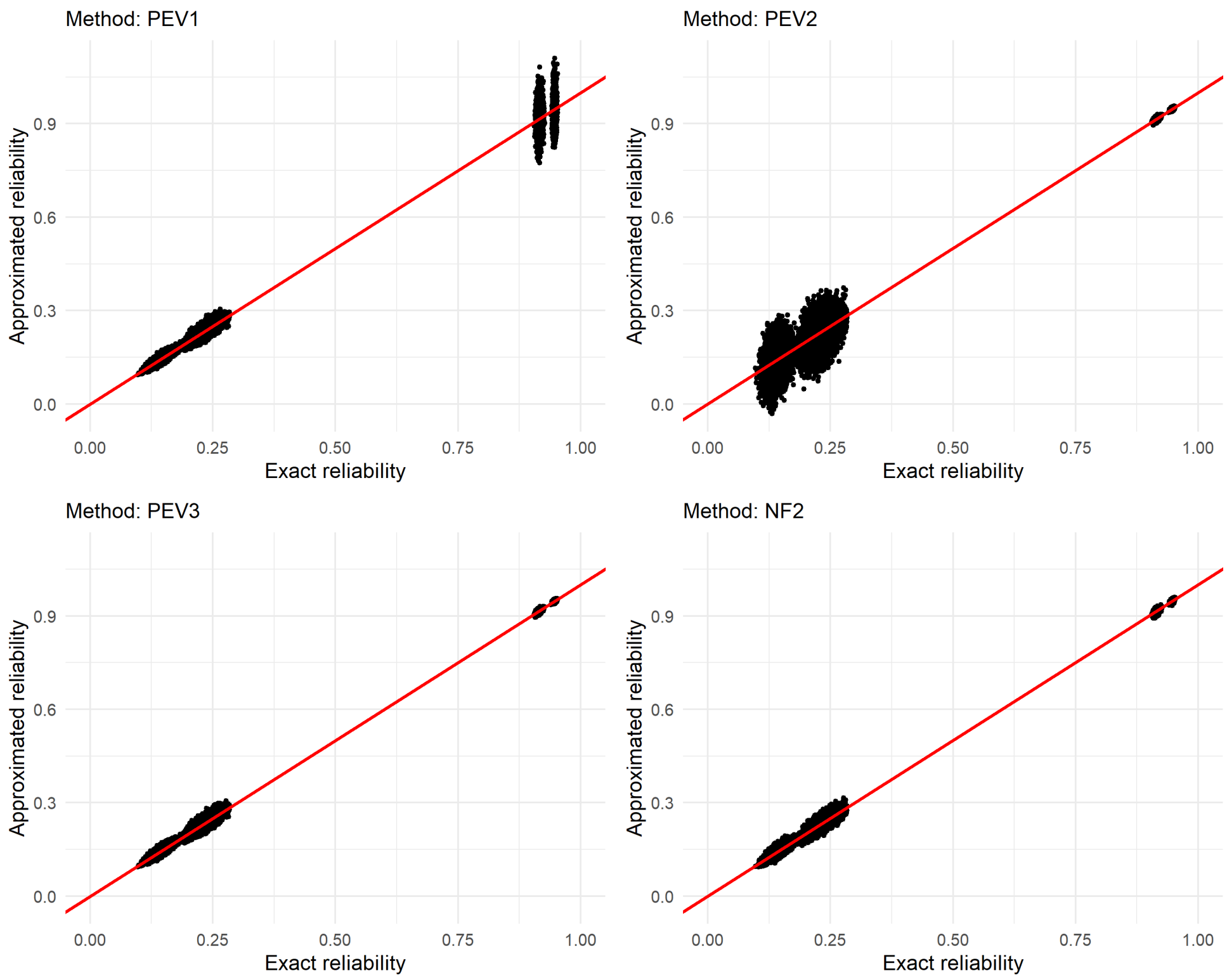

Fig. S1.3: Scatterplots with exact reliability on the x-axis and approximated reliability on the y-axis. Number of MC samples: 1,000. The red line represents $y = x$

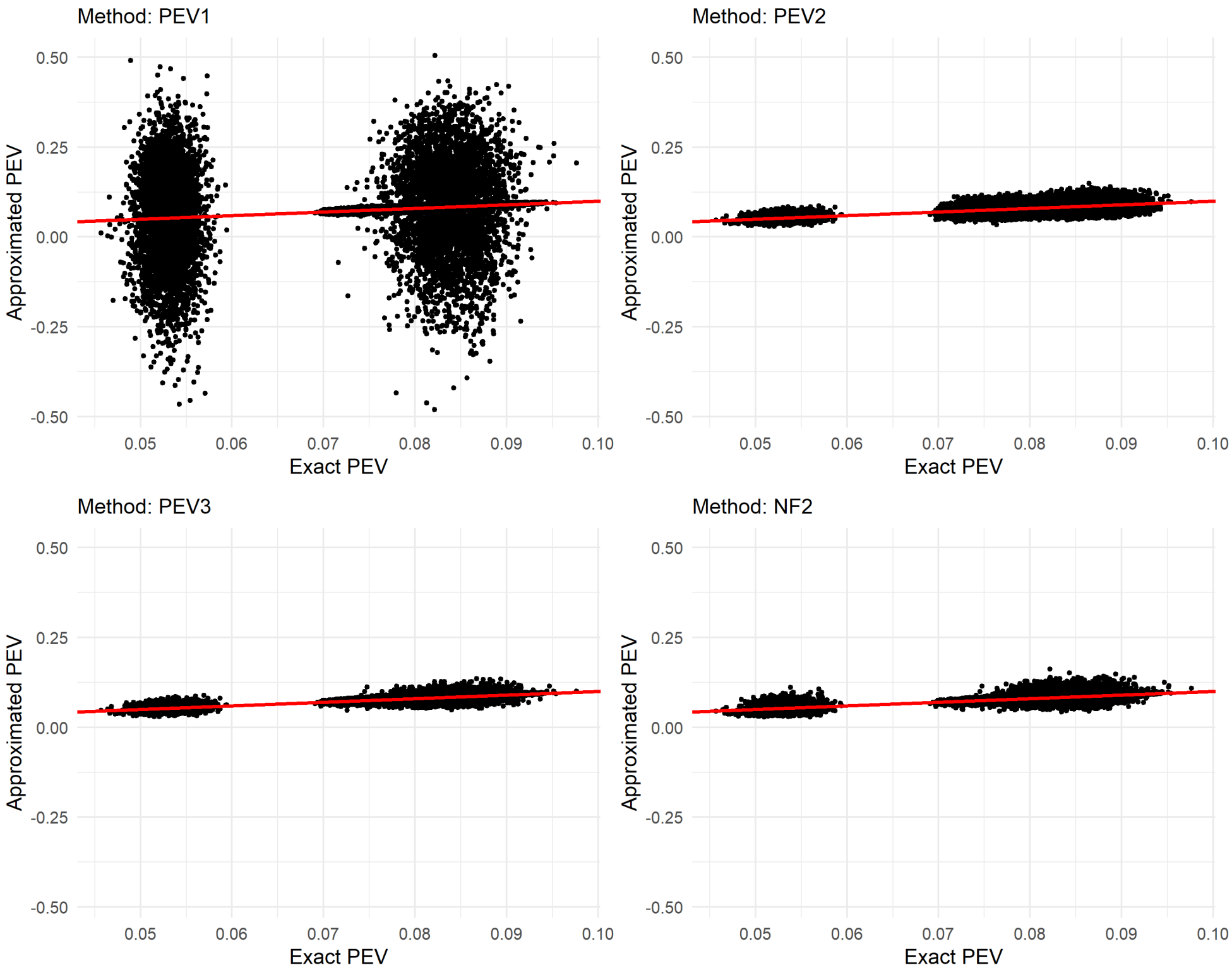

Fig. S1.4: Scatterplots with exact PEV on the x-axis and approximated PEV on the y-axis. Number of MC samples: 100. The red line represents $y = x$

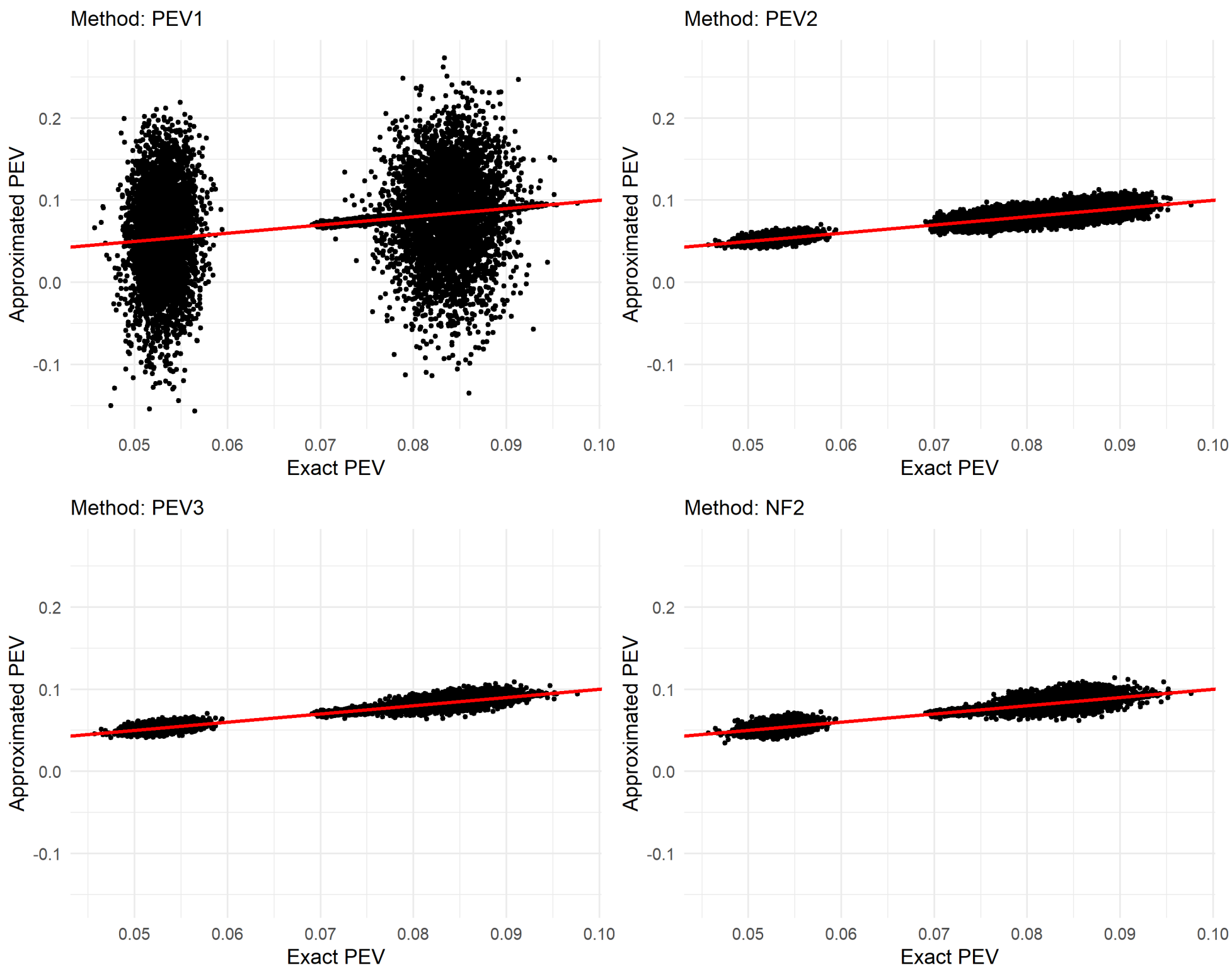

Fig. S1.5: Scatterplots with exact PEV on the x-axis and approximated PEV on the y-axis. Number of MC samples: 500. The red line represents $y = x$

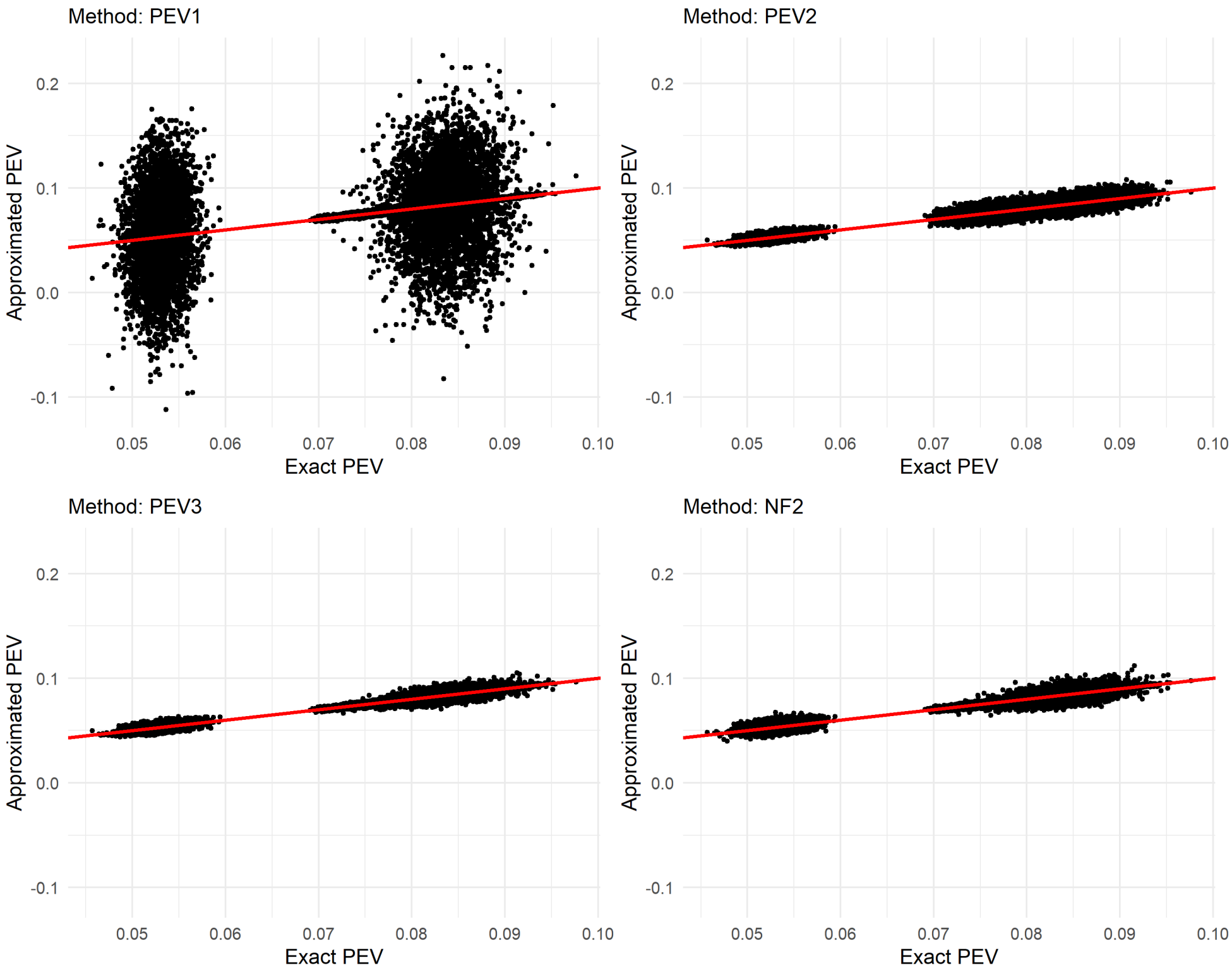

Fig. S1.6: Scatterplots with exact PEV on the x-axis and approximated PEV on the y-axis. Number of MC samples: 1,000. The red line represents $y = x$
