# Supplementary material for: Approximating prediction error variances and reliabilities in a multiple-trait genomic prediction model using Monte Carlo sampling
Source: Theor Appl Genet. 2026 Aug 28;139(9):250. doi: 10.1007/s00122-026-05336-0 (PMC13525033; doi:10.1007/s00122-026-05336-0)
Supplement: Supplementary file 2 [file 122_2026_5336_MOESM2_ESM.pdf]

Supplementary material 2 for: Approximating prediction error variances and reliabilities in multiple-trait genomic prediction model using Monte Carlo sampling

Antero Heikkilä[1*], Ismo Strandén[2], Martin Lidauer[2], Klaus Nordhausen[3], Sara Taskinen[1]

[1*]Department of Mathematics and Statistics, University of Jyväskylä, Jyväskylä, FI-40014, Finland.
[2] Natural Resources Institute Finland (Luke), Jokioinen, FI-31600, Finland.
[3]Department of Mathematics and Statistics, University of Helsinki, Helsinki, FI-00014, Finland.

*Corresponding author(s). E-mail(s): antero.j.heikkila@jyu.fi;
Contributing authors: ismo.stranden@luke.fi ; martin.lidauer@luke.fi ; klaus.nordhausen@helsinki.fi; sara.taskinen@jyu.fi;

This supplementary material contains results related to the different setting, described below. This setting differs from the original setting described in the main article. We performed the analysis using three MC sample sizes: 100, 500, and 1,000. Results related to reliability approximation are presented in Tables S2.1–S2.3 and S2.7–S2.9, and in Figures S2.1-S2.3. Results related to PEV approximation are presented in Tables S2.4–S2.6 and S2.10–S2.12, and in Figures S2.4–S2.6.

Here, the simulation and analysis is performed using a different set of simulation parameters. The number of individuals is the same: 1,000 individuals in parent pools 1 and 2. Parents produce 10,000 crosses. The genetic covariance matrices are as follows:

$$\mathbf{G_{0,1}} = \begin{bmatrix} 0.50 & 0.20 & -0.30 & -0.20 \\ 0.20 & 0.50 & -0.20 & -0.30 \\ -0.30 & -0.20 & 2.00 & 1.00 \\ -0.20 & -0.30 & 1.00 & 2.00 \end{bmatrix}, \mathbf{G_{0,2}} = \begin{bmatrix} 0.10 & 0.08 & -0.42 & -0.28 \\ 0.08 & 0.10 & -0.28 & -0.42 \\ -0.42 & -0.28 & 20.00 & 14.00 \\ -0.28 & -0.42 & 14.00 & 20.00 \end{bmatrix} \text{ and}$$

$$\mathbf{G_{0,3}} = \begin{bmatrix} 0.10 & 0.05 & 0.00 & -0.07 \\ 0.05 & 0.10 & -0.07 & -0.04 \\ 0.00 & -0.07 & 0.20 & 0.14 \\ -0.07 & -0.04 & 0.14 & 0.20 \end{bmatrix}.$$

Residuals were assumed to be uncorrelated across character-states, yielding the residual covariance matrix:

$$\mathbf{R_0} = \begin{bmatrix} 1 & 0 & 0 & 0 \\ 0 & 5 & 0 & 0 \\ 0 & 0 & 50 & 0 \\ 0 & 0 & 0 & 100 \end{bmatrix}.$$

In both the genetic and residual covariance matrices, the first two rows and columns correspond to trait A in the two locations, and the last two to trait B in the two locations. Off-diagonal elements denote covariances.

Table S2.1: Comparison of different methods approximating the exact reliability and their estimates with different statistics using 100 MC samples

Statistic	PEV1	PEV2	PEV3	NF2
Correlation	0.975	0.939	0.994	0.993
RMSE	0.063	0.101	0.031	0.033
MAX	0.611	0.579	0.195	0.198
Intercept	0.018	0.041	0.003	0.003
Slope	0.946	0.882	0.989	0.989

Table S2.2: Comparison of different methods approximating the exact reliability and their estimates with different statistics using 500 MC samples

Statistic	PEV1	PEV2	PEV3	NF2
Correlation	0.995	0.987	0.999	0.999
RMSE	0.028	0.045	0.013	0.014
MAX	0.223	0.241	0.083	0.082
Intercept	0.004	0.009	0.001	0.001
Slope	0.990	0.975	0.998	0.998

Table S2.3: Comparison of different methods approximating the exact reliability and their estimates with different statistics using 1,000 MC samples

Statistic	PEV1	PEV2	PEV3	NF2
Correlation	0.997	0.993	0.999	0.999
RMSE	0.020	0.032	0.010	0.010
MAX	0.192	0.174	0.059	0.058
Intercept	0.002	0.004	0.000	0.000
Slope	0.995	0.988	0.999	0.999

Table S2.4: Comparison of different methods approximating the exact PEV and their estimates with different statistics using 100 MC samples

Statistic	PEV1	PEV2	PEV3	NF2
Correlation	0.185	0.980	0.986	0.978
RMSE	0.583	0.030	0.024	0.032
MAX	12.404	0.463	0.466	0.684
Intercept	0.141	0.006	0.004	0.007
Slope	0.046	0.959	0.969	0.948

Table S2.5: Comparison of different methods approximating the exact PEV and their estimates with different statistics using 500 MC samples

Statistic	PEV1	PEV2	PEV3	NF2
Correlation	0.448	0.996	0.997	0.995
RMSE	0.248	0.013	0.011	0.014
MAX	4.077	0.212	0.213	0.313
Intercept	0.114	0.001	0.001	0.001
Slope	0.236	0.991	0.994	0.993

Table S2.6: Comparison of different methods approximating the exact PEV and their estimates with different statistics using 1,000 MC samples

Statistic	PEV1	PEV2	PEV3	NF2
Correlation	0.617	0.998	0.999	0.998
RMSE	0.177	0.009	0.008	0.010
MAX	3.886	0.154	0.150	0.212
Intercept	0.089	0.001	0.000	0.001
Slope	0.401	0.996	0.997	0.996

Table S2.7: Comparison of different methods for approximating exact reliability and their estimates with different statistics using 100 MC samples

Statistic	Group	Trait	Range of r^2	PEV1	PEV2	PEV3	NF2
Correlation	parent pool 1	1	0.95-0.97	0.015	0.305	0.306	0.234
		2	0.90-0.92	0.042	0.263	0.260	0.205
		3	0.81-0.86	0.029	0.291	0.285	0.232
		4	0.74-0.81	0.017	0.290	0.278	0.219
	parent pool 2	1	0.85-0.89	0.112	0.337	0.343	0.307
		2	0.77-0.83	0.189	0.294	0.318	0.329
		3	0.97-0.98	0.011	0.204	0.204	0.150
		4	0.95-0.96	0.044	0.175	0.179	0.161
	hybrid	1	0.34-0.53	0.323	0.269	0.391	0.397
		2	0.19-0.31	0.384	0.135	0.397	0.362
		3	0.06-0.13	0.528	0.072	0.529	0.437
		4	0.12-0.18	0.337	0.063	0.339	0.281
RMSE	parent pool 1	1	0.95-0.97	0.135	0.005	0.005	0.007
		2	0.90-0.92	0.130	0.012	0.012	0.017
		3	0.81-0.86	0.111	0.023	0.023	0.027
		4	0.74-0.81	0.111	0.033	0.032	0.036
	parent pool 2	1	0.85-0.89	0.126	0.018	0.018	0.023
		2	0.77-0.83	0.122	0.028	0.027	0.032
		3	0.97-0.98	0.139	0.004	0.004	0.005
		4	0.95-0.96	0.146	0.006	0.006	0.008
	hybrid	1	0.34-0.53	0.065	0.076	0.050	0.050
		2	0.19-0.31	0.035	0.108	0.034	0.038
		3	0.06-0.13	0.013	0.129	0.013	0.017
		4	0.12-0.18	0.021	0.122	0.021	0.026
MAX	parent pool 1	1	0.95-0.97	0.522	0.017	0.017	0.029
		2	0.90-0.92	0.495	0.046	0.046	0.056
		3	0.81-0.86	0.364	0.080	0.092	0.125
		4	0.74-0.81	0.410	0.120	0.133	0.145
	parent pool 2	1	0.85-0.89	0.454	0.070	0.075	0.089
		2	0.77-0.83	0.507	0.113	0.104	0.117
		3	0.97-0.98	0.518	0.013	0.013	0.025
		4	0.95-0.96	0.611	0.023	0.023	0.033
	hybrid	1	0.34-0.53	0.305	0.326	0.195	0.198
		2	0.19-0.31	0.165	0.467	0.175	0.178
		3	0.06-0.13	0.065	0.579	0.067	0.090
		4	0.12-0.18	0.082	0.532	0.112	0.126
Intercept	parent pool 1	1	0.95-0.97	0.962	0.878	0.878	0.914
		2	0.90-0.92	0.908	0.842	0.843	0.870
		3	0.81-0.86	0.837	0.767	0.769	0.790
		4	0.74-0.81	0.773	0.704	0.706	0.725
	parent pool 2	1	0.85-0.89	0.870	0.786	0.784	0.809
		2	0.77-0.83	0.794	0.734	0.727	0.735
		3	0.97-0.98	0.974	0.930	0.930	0.950
		4	0.95-0.96	0.956	0.917	0.917	0.929
	hybrid	1	0.34-0.53	0.414	0.427	0.390	0.388
		2	0.19-0.31	0.214	0.245	0.212	0.218
		3	0.06-0.13	0.066	0.090	0.066	0.074
		4	0.12-0.18	0.134	0.149	0.133	0.138
Slope	parent pool 1	1	0.95-0.97	0.000	0.088	0.088	0.050
		2	0.90-0.92	0.001	0.074	0.073	0.043
		3	0.81-0.86	0.002	0.086	0.084	0.059
		4	0.74-0.81	0.002	0.091	0.089	0.064
	parent pool 2	1	0.85-0.89	0.005	0.101	0.103	0.075
		2	0.77-0.83	0.014	0.089	0.098	0.087
		3	0.97-0.98	0.000	0.045	0.045	0.024
		4	0.95-0.96	0.000	0.041	0.041	0.028
	hybrid	1	0.34-0.53	0.100	0.072	0.152	0.155
		2	0.19-0.31	0.140	0.017	0.149	0.125
		3	0.06-0.13	0.274	0.004	0.273	0.183
		4	0.12-0.18	0.109	0.004	0.109	0.076

Table S2.8: Comparison of different methods for approximating exact reliability and their estimates with different statistics using 500 MC samples

Statistic	Group	Trait	Range of r^2	PEV1	PEV2	PEV3	NF2
Correlation	parent pool 1	1	0.95-0.97	-0.027	0.601	0.600	0.455
		2	0.90-0.92	0.102	0.546	0.548	0.460
		3	0.81-0.86	0.161	0.542	0.549	0.491
		4	0.74-0.81	0.226	0.587	0.601	0.559
	parent pool 2	1	0.85-0.89	0.132	0.602	0.609	0.538
		2	0.77-0.83	0.232	0.551	0.573	0.544
		3	0.97-0.98	0.015	0.464	0.464	0.366
		4	0.95-0.96	0.059	0.426	0.428	0.354
	hybrid	1	0.34-0.53	0.598	0.543	0.702	0.703
		2	0.19-0.31	0.665	0.293	0.686	0.648
		3	0.06-0.13	0.809	0.155	0.811	0.733
		4	0.12-0.18	0.616	0.147	0.623	0.551
RMSE	parent pool 1	1	0.95-0.97	0.063	0.002	0.002	0.003
		2	0.90-0.92	0.056	0.006	0.006	0.007
		3	0.81-0.86	0.051	0.010	0.010	0.012
		4	0.74-0.81	0.048	0.014	0.014	0.016
	parent pool 2	1	0.85-0.89	0.054	0.008	0.008	0.010
		2	0.77-0.83	0.050	0.013	0.012	0.014
		3	0.97-0.98	0.059	0.002	0.002	0.002
		4	0.95-0.96	0.062	0.003	0.003	0.004
	hybrid	1	0.34-0.53	0.029	0.034	0.022	0.022
		2	0.19-0.31	0.016	0.047	0.015	0.017
		3	0.06-0.13	0.006	0.057	0.006	0.007
		4	0.12-0.18	0.009	0.053	0.009	0.011
MAX	parent pool 1	1	0.95-0.97	0.203	0.009	0.010	0.014
		2	0.90-0.92	0.182	0.017	0.017	0.026
		3	0.81-0.86	0.223	0.041	0.040	0.047
		4	0.74-0.81	0.181	0.046	0.049	0.058
	parent pool 2	1	0.85-0.89	0.161	0.028	0.030	0.037
		2	0.77-0.83	0.186	0.043	0.052	0.064
		3	0.97-0.98	0.204	0.006	0.006	0.007
		4	0.95-0.96	0.204	0.011	0.011	0.015
	hybrid	1	0.34-0.53	0.108	0.137	0.083	0.082
		2	0.19-0.31	0.069	0.179	0.065	0.074
		3	0.06-0.13	0.022	0.241	0.021	0.032
		4	0.12-0.18	0.037	0.219	0.038	0.048
Intercept	parent pool 1	1	0.95-0.97	0.963	0.651	0.651	0.774
		2	0.90-0.92	0.903	0.638	0.638	0.726
		3	0.81-0.86	0.821	0.578	0.573	0.632
		4	0.74-0.81	0.736	0.504	0.491	0.531
	parent pool 2	1	0.85-0.89	0.862	0.571	0.567	0.641
		2	0.77-0.83	0.773	0.547	0.532	0.572
		3	0.97-0.98	0.974	0.767	0.767	0.845
		4	0.95-0.96	0.955	0.763	0.762	0.832
	hybrid	1	0.34-0.53	0.297	0.328	0.237	0.237
		2	0.19-0.31	0.139	0.229	0.132	0.145
		3	0.06-0.13	0.031	0.089	0.030	0.042
		4	0.12-0.18	0.094	0.147	0.092	0.105
Slope	parent pool 1	1	0.95-0.97	-0.001	0.324	0.324	0.197
		2	0.90-0.92	0.007	0.298	0.299	0.202
		3	0.81-0.86	0.022	0.311	0.317	0.247
		4	0.74-0.81	0.049	0.350	0.366	0.315
	parent pool 2	1	0.85-0.89	0.014	0.346	0.352	0.268
		2	0.77-0.83	0.040	0.321	0.340	0.290
		3	0.97-0.98	0.000	0.212	0.212	0.132
		4	0.95-0.96	0.001	0.202	0.203	0.129
	hybrid	1	0.34-0.53	0.354	0.286	0.485	0.485
		2	0.19-0.31	0.444	0.083	0.470	0.417
		3	0.06-0.13	0.663	0.021	0.664	0.536
		4	0.12-0.18	0.376	0.020	0.383	0.297

Table S2.9: Comparison of different methods for approximating exact reliability and their estimates with different statistics using 1,000 MC samples

Statistic	Group	Trait	Range of r^2	PEV1	PEV2	PEV3	NF2
Correlation	parent pool 1	1	0.95-0.97	-0.059	0.716	0.715	0.568
		2	0.90-0.92	0.121	0.653	0.656	0.573
		3	0.81-0.86	0.202	0.687	0.692	0.628
		4	0.74-0.81	0.284	0.724	0.738	0.695
	parent pool 2	1	0.85-0.89	0.196	0.736	0.743	0.684
		2	0.77-0.83	0.285	0.704	0.721	0.683
		3	0.97-0.98	0.049	0.582	0.583	0.489
		4	0.95-0.96	0.048	0.577	0.577	0.469
	hybrid	1	0.34-0.53	0.721	0.666	0.810	0.810
		2	0.19-0.31	0.784	0.386	0.800	0.766
		3	0.06-0.13	0.888	0.203	0.889	0.834
		4	0.12-0.18	0.739	0.194	0.744	0.676
RMSE	parent pool 1	1	0.95-0.97	0.043	0.002	0.002	0.002
		2	0.90-0.92	0.039	0.004	0.004	0.005
		3	0.81-0.86	0.036	0.007	0.007	0.008
		4	0.74-0.81	0.035	0.010	0.010	0.011
	parent pool 2	1	0.85-0.89	0.038	0.006	0.006	0.007
		2	0.77-0.83	0.037	0.009	0.009	0.010
		3	0.97-0.98	0.043	0.001	0.001	0.002
		4	0.95-0.96	0.043	0.002	0.002	0.003
	hybrid	1	0.34-0.53	0.020	0.024	0.015	0.015
		2	0.19-0.31	0.011	0.034	0.011	0.012
		3	0.06-0.13	0.004	0.040	0.004	0.005
		4	0.12-0.18	0.007	0.038	0.007	0.008
MAX	parent pool 1	1	0.95-0.97	0.137	0.005	0.005	0.007
		2	0.90-0.92	0.131	0.014	0.014	0.016
		3	0.81-0.86	0.114	0.023	0.022	0.029
		4	0.74-0.81	0.116	0.036	0.036	0.041
	parent pool 2	1	0.85-0.89	0.130	0.020	0.020	0.025
		2	0.77-0.83	0.127	0.028	0.029	0.033
		3	0.97-0.98	0.190	0.004	0.004	0.005
		4	0.95-0.96	0.192	0.008	0.008	0.011
	hybrid	1	0.34-0.53	0.080	0.092	0.059	0.058
		2	0.19-0.31	0.052	0.135	0.049	0.053
		3	0.06-0.13	0.020	0.174	0.020	0.022
		4	0.12-0.18	0.025	0.151	0.025	0.031
Intercept	parent pool 1	1	0.95-0.97	0.965	0.501	0.501	0.647
		2	0.90-0.92	0.899	0.508	0.506	0.610
		3	0.81-0.86	0.806	0.423	0.418	0.496
		4	0.74-0.81	0.709	0.373	0.354	0.398
	parent pool 2	1	0.85-0.89	0.848	0.424	0.418	0.497
		2	0.77-0.83	0.751	0.405	0.387	0.439
		3	0.97-0.98	0.973	0.645	0.645	0.751
		4	0.95-0.96	0.955	0.623	0.623	0.739
	hybrid	1	0.34-0.53	0.221	0.260	0.160	0.160
		2	0.19-0.31	0.096	0.212	0.090	0.103
		3	0.06-0.13	0.019	0.087	0.019	0.027
		4	0.12-0.18	0.068	0.144	0.067	0.082
Slope	parent pool 1	1	0.95-0.97	-0.002	0.479	0.479	0.328
		2	0.90-0.92	0.011	0.441	0.443	0.329
		3	0.81-0.86	0.039	0.496	0.502	0.409
		4	0.74-0.81	0.084	0.518	0.543	0.486
	parent pool 2	1	0.85-0.89	0.030	0.515	0.522	0.432
		2	0.77-0.83	0.067	0.497	0.519	0.454
		3	0.97-0.98	0.001	0.337	0.338	0.228
		4	0.95-0.96	0.002	0.348	0.349	0.227
	hybrid	1	0.34-0.53	0.519	0.435	0.652	0.651
		2	0.19-0.31	0.616	0.149	0.640	0.587
		3	0.06-0.13	0.793	0.039	0.794	0.695
		4	0.12-0.18	0.545	0.037	0.552	0.454

Table S2.10: Comparison of different methods for approximating exact PEV and their estimates with different statistics using 100 MC samples

Statistic	Group	Trait	Range of PEV	PEV1	PEV2	PEV3	NF2
Correlation	parent pool 1	1	0.02-0.02	0.050	0.334	0.336	0.280
		2	0.04-0.05	0.021	0.282	0.277	0.207
		3	0.27-0.38	-0.009	0.343	0.330	0.250
		4	0.38-0.52	0.022	0.337	0.319	0.254
	parent pool 2	1	0.01-0.02	0.142	0.375	0.385	0.352
		2	0.02-0.02	0.201	0.354	0.376	0.378
		3	0.48-0.60	0.054	0.202	0.203	0.178
		4	0.78-0.97	0.112	0.220	0.226	0.241
	hybrid	1	0.05-0.07	0.390	0.329	0.471	0.476
		2	0.06-0.08	0.621	0.266	0.636	0.602
		3	0.16-0.20	0.918	0.233	0.917	0.871
		4	0.15-0.19	0.792	0.230	0.791	0.728
RMSE	parent pool 1	1	0.02-0.02	0.068	0.003	0.003	0.004
		2	0.04-0.05	0.065	0.006	0.006	0.008
		3	0.27-0.38	0.221	0.046	0.046	0.055
		4	0.38-0.52	0.222	0.065	0.064	0.072
	parent pool 2	1	0.01-0.02	0.013	0.002	0.002	0.002
		2	0.02-0.02	0.012	0.003	0.003	0.003
		3	0.48-0.60	2.778	0.074	0.074	0.104
		4	0.78-0.97	2.910	0.126	0.126	0.169
	hybrid	1	0.05-0.07	0.007	0.008	0.005	0.005
		2	0.06-0.08	0.004	0.011	0.003	0.004
		3	0.16-0.20	0.003	0.026	0.003	0.003
		4	0.15-0.19	0.004	0.024	0.004	0.005
MAX	parent pool 1	1	0.02-0.02	0.259	0.008	0.008	0.015
		2	0.04-0.05	0.248	0.022	0.021	0.028
		3	0.27-0.38	0.761	0.164	0.191	0.252
		4	0.38-0.52	0.832	0.240	0.264	0.288
	parent pool 2	1	0.01-0.02	0.045	0.007	0.007	0.009
		2	0.02-0.02	0.051	0.012	0.011	0.012
		3	0.48-0.60	10.623	0.257	0.257	0.486
		4	0.78-0.97	12.404	0.463	0.466	0.684
	hybrid	1	0.05-0.07	0.030	0.033	0.019	0.020
		2	0.06-0.08	0.017	0.046	0.018	0.018
		3	0.16-0.20	0.013	0.120	0.014	0.018
		4	0.15-0.19	0.017	0.112	0.023	0.026
Intercept	parent pool 1	1	0.02-0.02	0.019	0.017	0.017	0.017
		2	0.04-0.05	0.045	0.041	0.042	0.043
		3	0.27-0.38	0.322	0.287	0.288	0.299
		4	0.38-0.52	0.450	0.398	0.400	0.414
	parent pool 2	1	0.01-0.02	0.012	0.011	0.011	0.011
		2	0.02-0.02	0.019	0.017	0.017	0.017
		3	0.48-0.60	0.524	0.501	0.501	0.510
		4	0.78-0.97	0.877	0.830	0.829	0.839
	hybrid	1	0.05-0.07	0.046	0.048	0.042	0.042
		2	0.06-0.08	0.047	0.070	0.046	0.049
		3	0.16-0.20	0.029	0.172	0.029	0.045
		4	0.15-0.19	0.064	0.162	0.065	0.082
Slope	parent pool 1	1	0.02-0.02	0.001	0.103	0.103	0.064
		2	0.04-0.05	0.001	0.085	0.083	0.047
		3	0.27-0.38	-0.001	0.109	0.105	0.070
		4	0.38-0.52	0.002	0.117	0.113	0.082
	parent pool 2	1	0.01-0.02	0.008	0.133	0.136	0.101
		2	0.02-0.02	0.018	0.129	0.139	0.121
		3	0.48-0.60	0.000	0.044	0.044	0.028
		4	0.78-0.97	0.001	0.054	0.055	0.044
	hybrid	1	0.05-0.07	0.144	0.107	0.216	0.220
		2	0.06-0.08	0.376	0.065	0.392	0.348
		3	0.16-0.20	0.841	0.052	0.839	0.755
		4	0.15-0.19	0.621	0.050	0.619	0.522

Table S2.11: Comparison of different methods for approximating exact PEV and their estimates with different statistics using 500 MC samples

**Statistic**	**Group**	**Trait**	**Range of PEV**	**PEV1**	**PEV2**	**PEV3**	**NF2**
Correlation	parent pool 1	1	0.02-0.02	-0.041	0.619	0.618	0.466
		2	0.04-0.05	0.073	0.566	0.565	0.462
		3	0.27-0.38	0.177	0.595	0.602	0.542
		4	0.38-0.52	0.238	0.644	0.656	0.610
	parent pool 2	1	0.01-0.02	0.143	0.666	0.672	0.597
		2	0.02-0.02	0.246	0.637	0.655	0.616
		3	0.48-0.60	0.040	0.460	0.461	0.378
		4	0.78-0.97	0.076	0.452	0.455	0.385
	hybrid	1	0.05-0.07	0.674	0.618	0.769	0.770
		2	0.06-0.08	0.868	0.521	0.880	0.859
		3	0.16-0.20	0.983	0.475	0.983	0.971
		4	0.15-0.19	0.945	0.460	0.947	0.924
RMSE	parent pool 1	1	0.02-0.02	0.032	0.001	0.001	0.002
		2	0.04-0.05	0.028	0.003	0.003	0.004
		3	0.27-0.38	0.103	0.021	0.020	0.024
		4	0.38-0.52	0.096	0.029	0.028	0.031
	parent pool 2	1	0.01-0.02	0.005	0.001	0.001	0.001
		2	0.02-0.02	0.005	0.001	0.001	0.001
		3	0.48-0.60	1.181	0.033	0.033	0.044
		4	0.78-0.97	1.243	0.057	0.057	0.076
	hybrid	1	0.05-0.07	0.003	0.003	0.002	0.002
		2	0.06-0.08	0.002	0.005	0.001	0.002
		3	0.16-0.20	0.001	0.011	0.001	0.001
		4	0.15-0.19	0.002	0.011	0.002	0.002
MAX	parent pool 1	1	0.02-0.02	0.101	0.005	0.005	0.007
		2	0.04-0.05	0.091	0.008	0.008	0.012
		3	0.27-0.38	0.416	0.083	0.081	0.093
		4	0.38-0.52	0.375	0.093	0.100	0.113
	parent pool 2	1	0.01-0.02	0.016	0.003	0.003	0.004
		2	0.02-0.02	0.019	0.004	0.005	0.006
		3	0.48-0.60	4.011	0.115	0.115	0.137
		4	0.78-0.97	4.077	0.212	0.213	0.313
	hybrid	1	0.05-0.07	0.011	0.014	0.009	0.009
		2	0.06-0.08	0.007	0.018	0.007	0.008
		3	0.16-0.20	0.004	0.046	0.004	0.006
		4	0.15-0.19	0.008	0.042	0.008	0.010
Intercept	parent pool 1	1	0.02-0.02	0.019	0.012	0.012	0.015
		2	0.04-0.05	0.045	0.030	0.030	0.035
		3	0.27-0.38	0.314	0.206	0.204	0.229
		4	0.38-0.52	0.425	0.268	0.260	0.285
	parent pool 2	1	0.01-0.02	0.012	0.007	0.007	0.008
		2	0.02-0.02	0.018	0.011	0.011	0.012
		3	0.48-0.60	0.524	0.418	0.418	0.456
		4	0.78-0.97	0.876	0.679	0.678	0.748
	hybrid	1	0.05-0.07	0.030	0.034	0.022	0.022
		2	0.06-0.08	0.018	0.056	0.017	0.020
		3	0.16-0.20	0.006	0.142	0.006	0.011
		4	0.15-0.19	0.018	0.135	0.018	0.025
Slope	parent pool 1	1	0.02-0.02	-0.001	0.352	0.352	0.214
		2	0.04-0.05	0.005	0.328	0.328	0.219
		3	0.27-0.38	0.026	0.360	0.365	0.290
		4	0.38-0.52	0.058	0.407	0.424	0.369
	parent pool 2	1	0.01-0.02	0.018	0.429	0.435	0.339
		2	0.02-0.02	0.052	0.425	0.443	0.381
		3	0.48-0.60	0.001	0.203	0.203	0.131
		4	0.78-0.97	0.002	0.226	0.227	0.148
	hybrid	1	0.05-0.07	0.453	0.375	0.588	0.588
		2	0.06-0.08	0.754	0.258	0.770	0.728
		3	0.16-0.20	0.966	0.219	0.966	0.942
		4	0.15-0.19	0.892	0.207	0.894	0.851

Table S2.12: Comparison of different methods for approximating exact PEV and their estimates with different statistics using 1,000 MC samples

Statistic	Group	Trait	Range of PEV	PEV1	PEV2	PEV3	NF2
Correlation	parent pool 1	1	0.02-0.02	-0.039	0.737	0.736	0.599
		2	0.04-0.05	0.123	0.677	0.680	0.596
		3	0.27-0.38	0.237	0.728	0.734	0.676
		4	0.38-0.52	0.313	0.767	0.780	0.739
	parent pool 2	1	0.01-0.02	0.234	0.794	0.800	0.748
		2	0.02-0.02	0.312	0.777	0.790	0.752
		3	0.48-0.60	0.040	0.572	0.573	0.475
		4	0.78-0.97	0.017	0.610	0.610	0.486
	hybrid	1	0.05-0.07	0.787	0.739	0.861	0.861
		2	0.06-0.08	0.927	0.641	0.934	0.920
		3	0.16-0.20	0.991	0.602	0.991	0.985
		4	0.15-0.19	0.971	0.590	0.972	0.960
RMSE	parent pool 1	1	0.02-0.02	0.022	0.001	0.001	0.001
		2	0.04-0.05	0.020	0.002	0.002	0.003
		3	0.27-0.38	0.072	0.014	0.014	0.017
		4	0.38-0.52	0.070	0.021	0.020	0.022
	parent pool 2	1	0.01-0.02	0.004	0.001	0.001	0.001
		2	0.02-0.02	0.004	0.001	0.001	0.001
		3	0.48-0.60	0.856	0.024	0.024	0.032
		4	0.78-0.97	0.869	0.040	0.040	0.054
	hybrid	1	0.05-0.07	0.002	0.002	0.002	0.002
		2	0.06-0.08	0.001	0.003	0.001	0.001
		3	0.16-0.20	0.001	0.008	0.001	0.001
		4	0.15-0.19	0.001	0.008	0.001	0.002
MAX	parent pool 1	1	0.02-0.02	0.070	0.003	0.003	0.004
		2	0.04-0.05	0.066	0.007	0.007	0.008
		3	0.27-0.38	0.222	0.045	0.044	0.060
		4	0.38-0.52	0.231	0.073	0.073	0.082
	parent pool 2	1	0.01-0.02	0.013	0.002	0.002	0.002
		2	0.02-0.02	0.013	0.003	0.003	0.003
		3	0.48-0.60	3.783	0.079	0.078	0.095
		4	0.78-0.97	3.886	0.154	0.150	0.212
	hybrid	1	0.05-0.07	0.008	0.009	0.006	0.006
		2	0.06-0.08	0.005	0.014	0.005	0.005
		3	0.16-0.20	0.004	0.034	0.004	0.004
		4	0.15-0.19	0.005	0.031	0.005	0.006
Intercept	parent pool 1	1	0.02-0.02	0.019	0.009	0.009	0.012
		2	0.04-0.05	0.045	0.024	0.023	0.029
		3	0.27-0.38	0.306	0.147	0.145	0.175
		4	0.38-0.52	0.406	0.193	0.182	0.207
	parent pool 2	1	0.01-0.02	0.012	0.005	0.005	0.006
		2	0.02-0.02	0.018	0.008	0.007	0.009
		3	0.48-0.60	0.524	0.355	0.355	0.411
		4	0.78-0.97	0.877	0.541	0.541	0.659
	hybrid	1	0.05-0.07	0.020	0.025	0.014	0.014
		2	0.06-0.08	0.010	0.045	0.010	0.012
		3	0.16-0.20	0.003	0.117	0.003	0.006
		4	0.15-0.19	0.009	0.112	0.009	0.014
Slope	parent pool 1	1	0.02-0.02	-0.002	0.514	0.514	0.362
		2	0.04-0.05	0.012	0.481	0.483	0.362
		3	0.27-0.38	0.050	0.545	0.551	0.458
		4	0.38-0.52	0.103	0.573	0.598	0.543
	parent pool 2	1	0.01-0.02	0.042	0.598	0.604	0.516
		2	0.02-0.02	0.090	0.602	0.623	0.558
		3	0.48-0.60	0.001	0.323	0.323	0.216
		4	0.78-0.97	0.001	0.383	0.383	0.249
	hybrid	1	0.05-0.07	0.623	0.538	0.741	0.740
		2	0.06-0.08	0.861	0.402	0.871	0.842
		3	0.16-0.20	0.983	0.356	0.983	0.970
		4	0.15-0.19	0.944	0.344	0.946	0.920

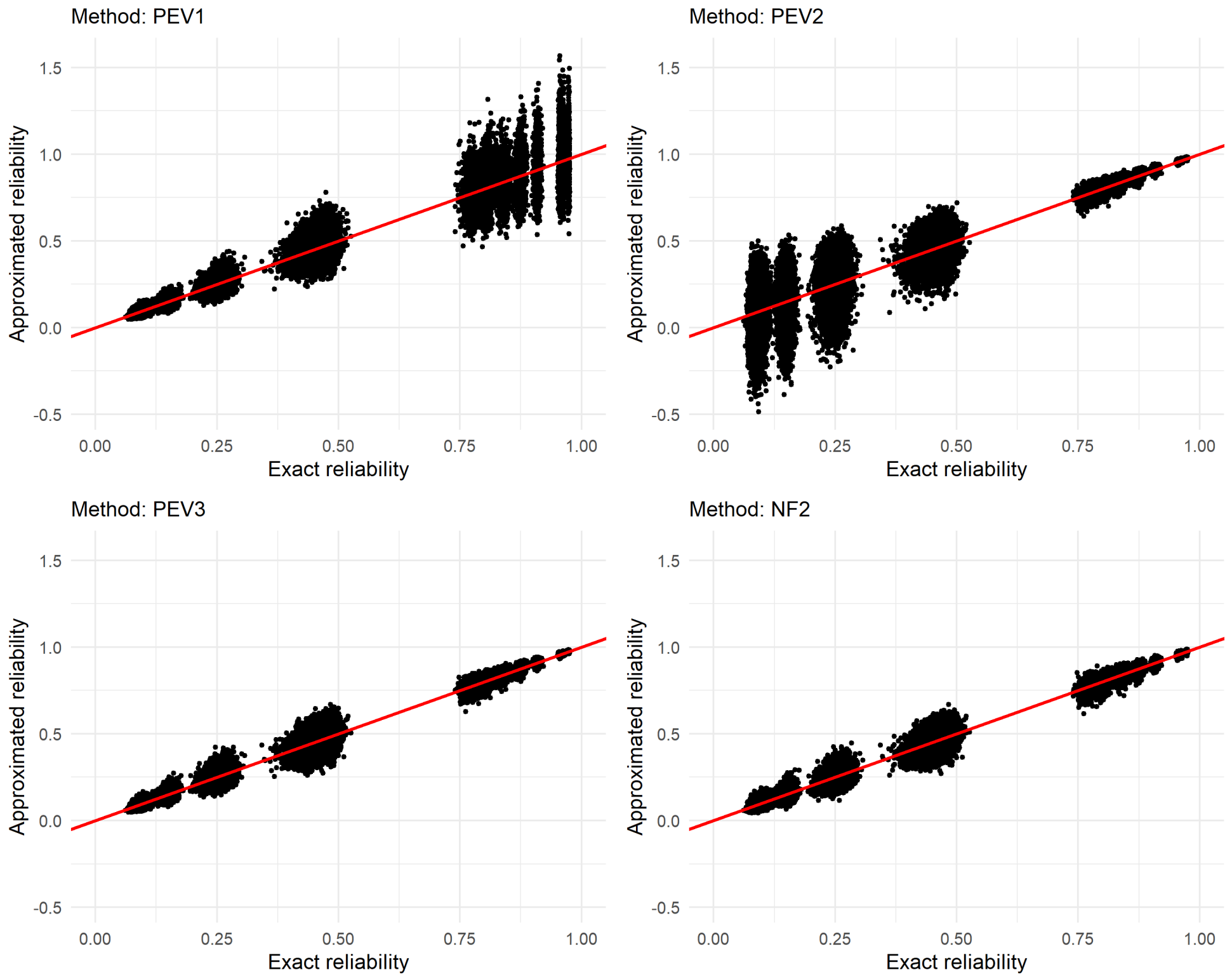

Fig. S2.1: Scatterplots with exact reliability on the x-axis and approximated reliability on the y-axis. Number of MC samples: 100. The red line represents $y = x$

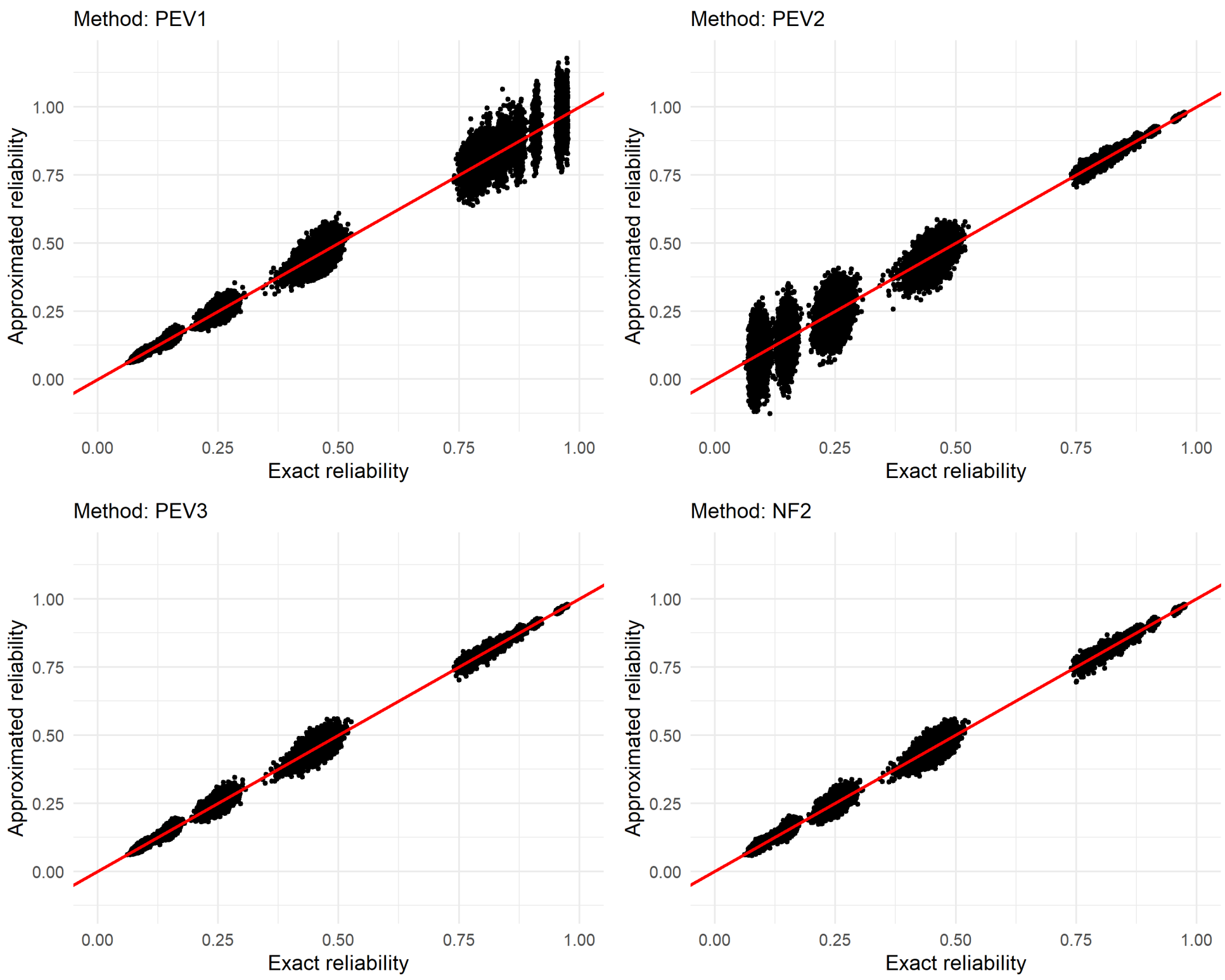

Fig. S2.2: Scatterplots with exact reliability on the x-axis and approximated reliability on the y-axis. Number of MC samples: 500. The red line represents $y = x$

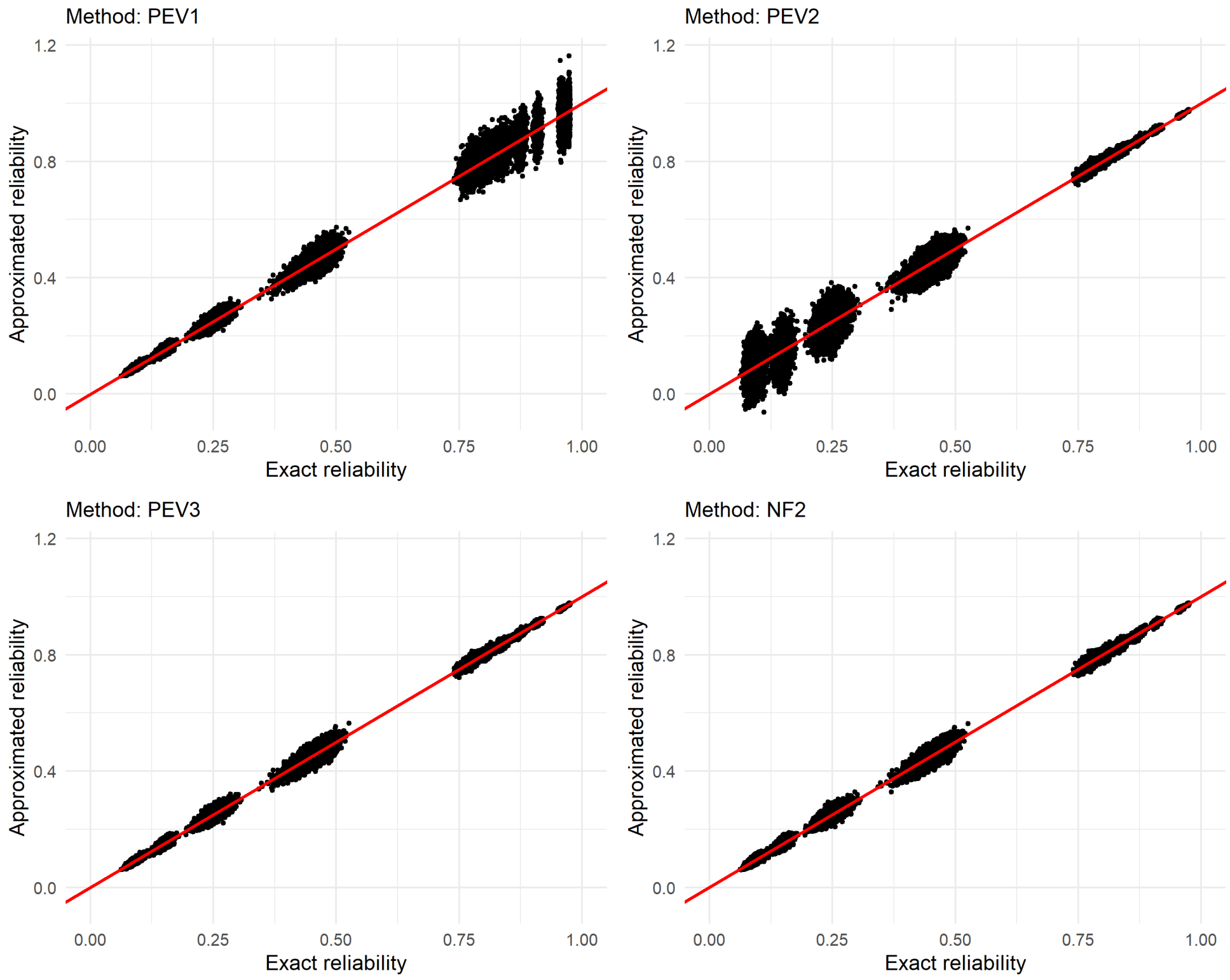

Fig. S2.3: Scatterplots with exact reliability on the x-axis and approximated reliability on the y-axis. Number of MC samples: 1,000. The red line represents $y = x$

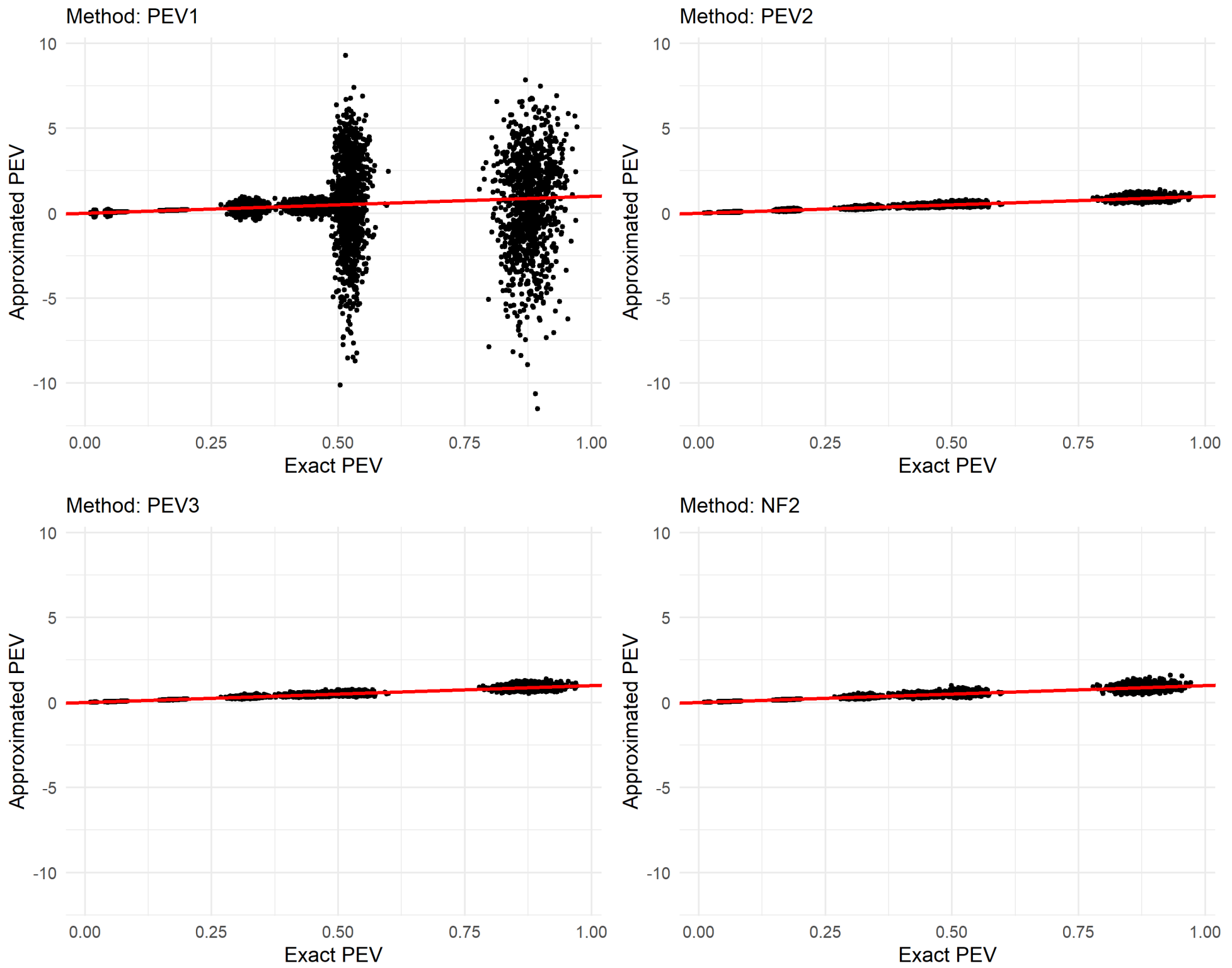

Fig. S2.4: Scatterplots with exact PEV on the x-axis and approximated PEV on the y-axis. Number of MC samples: 100. The red line represents $y = x$

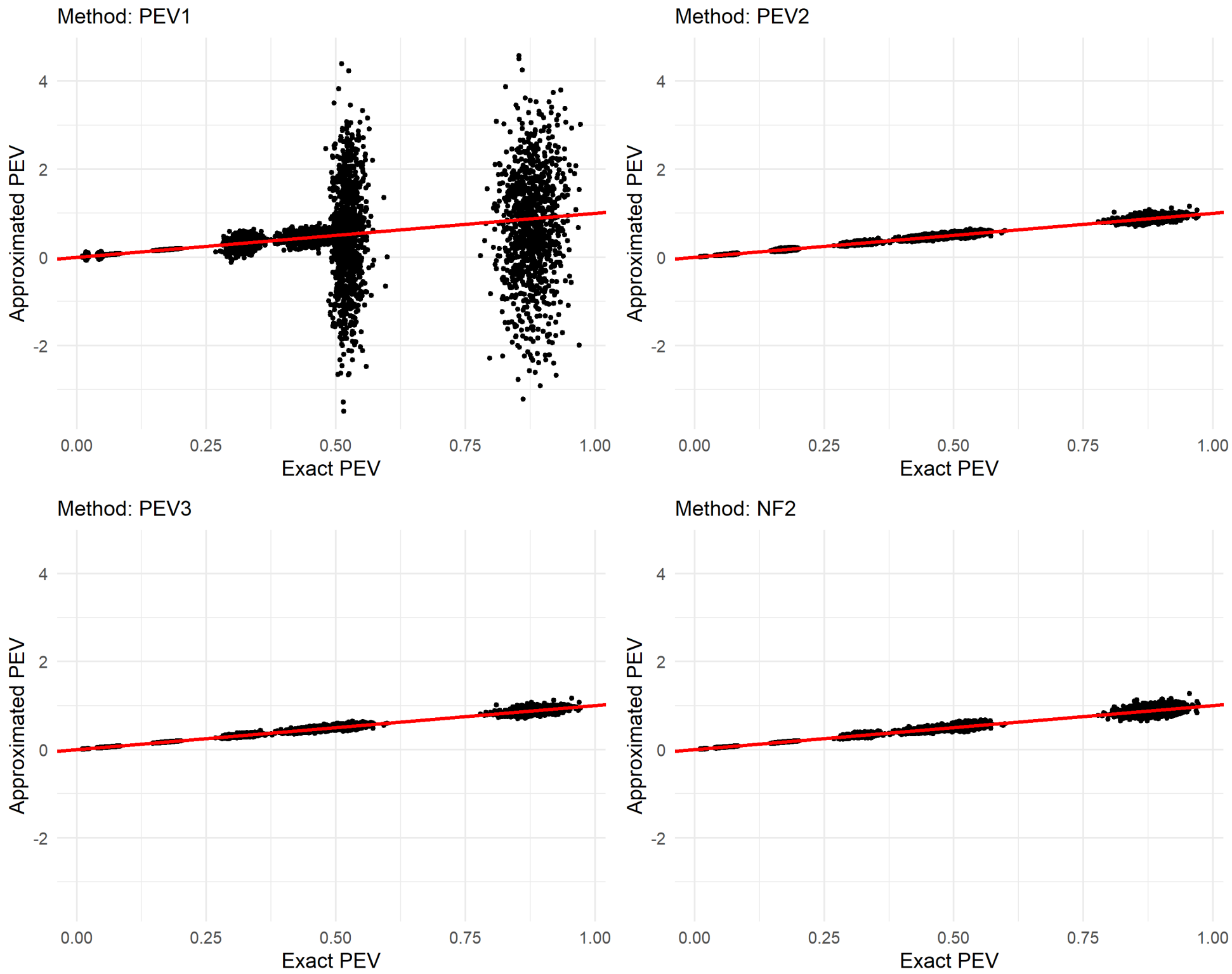

Fig. S2.5: Scatterplots with exact PEV on the x-axis and approximated PEV on the y-axis. Number of MC samples: 500. The red line represents $y = x$

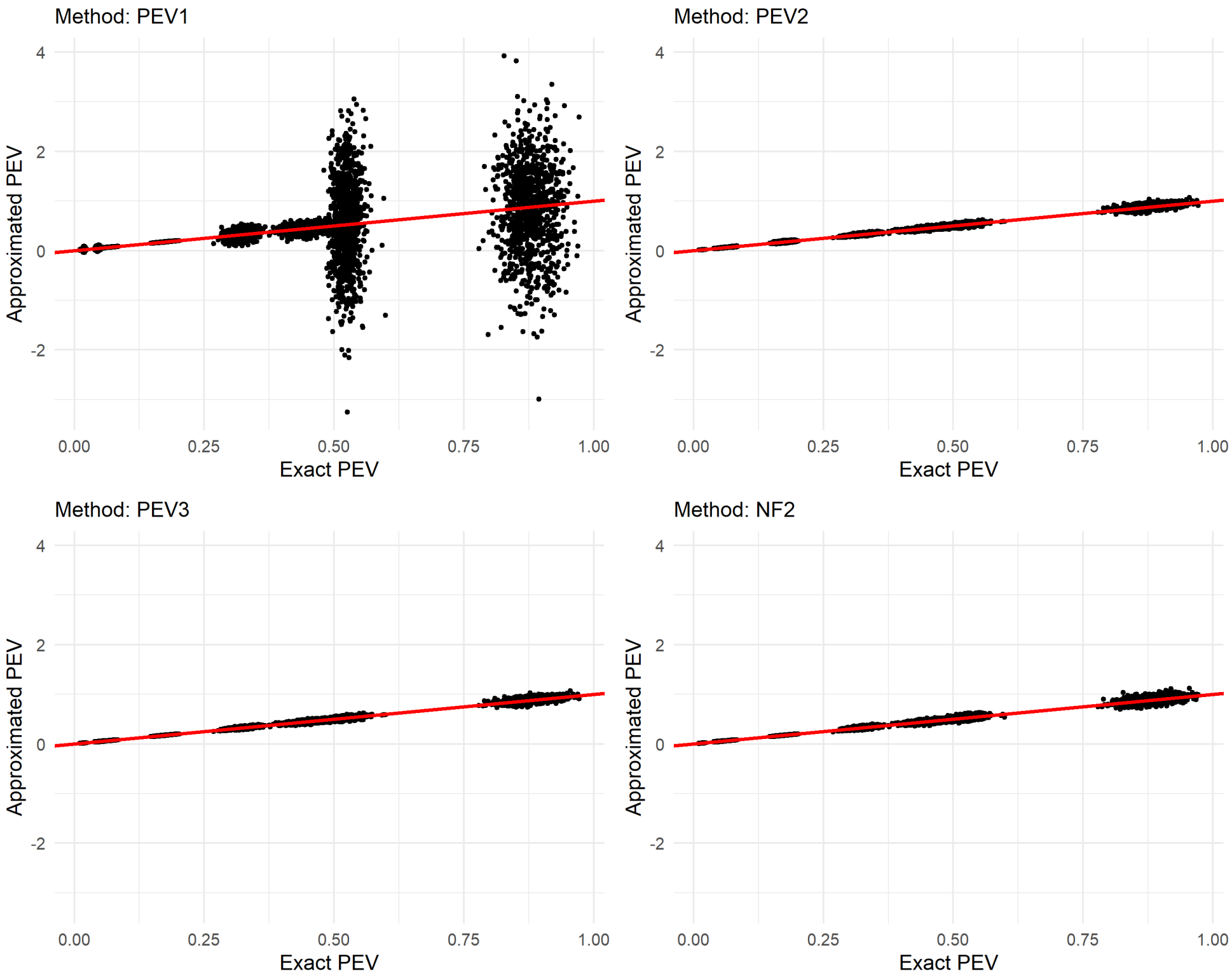

Fig. S2.6: Scatterplots with exact PEV on the x-axis and approximated PEV on the y-axis. Number of MC samples: 1,000. The red line represents $y = x$
